# Beyond Words: Speech Coordination Linked to Personality and Appraisals

**DOI:** 10.1007/s10919-025-00482-3

**Published:** 2025-03-08

**Authors:** Nicol A. Arellano-Véliz, Ramón D. Castillo, Bertus F. Jeronimus, E. Saskia Kunnen, Ralf F. A. Cox

**Affiliations:** 1 Department of Developmental Psychology, Faculty of Behavioral and Social Sciences, University of Groningen, Groningen, The Netherlands; 2 Laboratory of Cognitive Sciences, Faculty of Psychology, University of Talca, Talca, Chile

**Keywords:** Interpersonal coordination, Synchrony, Dyadic interactions, Personality, Speech coordination, Speech synchrony

## Abstract

We studied how personality differences and conversation topics predict interpersonal speech coordination, leading/following dynamics, and nonverbal interactional dominance in dyadic conversations. In a laboratory, 100 undergraduate students (50 same-gender dyads) had a 15-min conversation following three topics (introduction/self-disclosure/argumentation). Their speech coordination and turn-taking (speech/silence) dynamics were assessed through nonlinear time-series analyses: Cross-Recurrence Quantification Analysis (CRQA), Diagonal Cross-Recurrence Profiles (DCRP), and Anisotropic-CRQA. From the time series, we extracted five variables to operationalize speech coordination (global and at lag-zero), leading-following dynamics, and asymmetries in the interacting partners’ nonverbal interactional dominance. Interaction appraisals were also assessed. Associations between personality traits Extraversion/Agreeableness, speech coordination, and nonverbal interactional dominance were tested using mixed-effects models. Speech coordination and nonverbal interactional dominance differed across conversational topics and peaked during argumentative conversations. Extraversion was associated with increased speech coordination, and nonverbal interactional dominance, especially during the argumentative conversation. During a self-disclosure conversation, Extraversion concordance was associated with more symmetry in turn-taking dynamics. Speech coordination was generally associated with positive post-conversational appraisals such as wanting to meet in the future or liking the conversation partner, especially in extroverted individuals, whereas introverts seemed to value less swift dynamics. High Agreeableness predicted less speech coordination during argumentative conversations, and increased speech coordination (at lag-zero) predicted reduced perceived naturality in agreeable individuals. This may suggest a trade-off between maintaining swift speech dynamics and the natural flow of conversation for individuals high in Agreeableness.

**Supplementary Information:**

The online version contains supplementary material available at 10.1007/s10919-025-00482-3.

## Introduction

The course and dynamics of a conversation between two partners (a dyad) and how they experience the interaction are affected by contextual factors (i.e., situations) and individual differences (e.g., personality traits, see Harley, 2013). Interpersonal speech dynamics such as the temporal attunement of speech and silence turns show how dyads coordinate during conversations (through turn-taking dynamics), the leading-following dynamics they exhibit, and how potential nonverbal interactional dominance asymmetries emerge from the mutual influence of interacting partners. We present a study with four aims. First, we use complex dynamical systems theory and speech recordings during conversations to quantify differences in overall speech coordination through turn-taking behaviors, leader–follower dynamics (temporal domain), and differences or asymmetries in nonverbal interactional dominance between the interaction partners (e.g., one person tends to “dominate” the conversation to a greater extent through speech or silence episodes). Second, we examine whether these speech dynamics differ across three types of conversations, introduction, self-disclosure, and an argumentative conversation. Third, we examine how dyadic speech dynamics differ as a consequence of their personality traits, and study dyadic combinations (one or both low/high scores) of Extraversion (sociability) and Agreeableness (nurturance). Fourth, we examine how interpersonal speech coordination and personality traits influence how both interacting partners appraise their conversation regarding the perceived quality of the interaction and rapport. We conclude by discussing our study results and their fit into the broader interaction dynamics and personality theory literature.

### Interpersonal Speech Dynamics

Human communication extends beyond spoken words and comprises a complex flow of interpersonal dynamics within conversations. Language, viewed as a complex adaptive system, operates through interacting elements distributed across both the body and social environments. In this context, ‘body’ refers to the various physical and neural processes involved in language production and comprehension. This includes the motor functions necessary for speech, the sensory mechanism for hearing and understanding spoken language, and the cognitive processes that support language in communicative interaction (Di Paolo et al., 2018; Ellis & Larsen-Freeman, 2009; Lund et al., 2022). These processes modulate perceptions, emotions, and thoughts, which are transformed into meaningful language expressions (Scheidt et al., 2021). The behavior of each interacting partner is further influenced by characteristic adaptations—such as perceptual information, situational factors, social motivations, and other individual differences (Beckner et al., 2009; Asendorpf, 2017; Mischel & Shoda, 1995)—reflected in aspects like gaze, gestures, movement, and speech coordination (Fusaroli et al., 2014). Recognizing language as a complex adaptive system has significant implications for interpersonal interactions, where partners are linked through both verbal and nonverbal communication (Falandays et al., 2018; Scheidt et al., 2021; Thibault, 2004). These joint dynamics emerge in dyadic interactions, where both participants continuously shape the conversation as it unfolds (Reuzel et al., 2013), a crucial consideration given that dyads represent the majority of human social interactions (Peperkoorn et al., 2020).

In conversation, people adapt to each other through synchronized behaviors such as turn-taking, speech duration, speech rate, response latency, vocal intensity, and movement (Bloomfield et al., 2021; Fowler et al., 2008; Reuzel et al., 2014). This study focuses on three key aspects of interpersonal speech dynamics, in line with Reuzel et al. (2013, 2014): (1) Speech coordination, where a well-coordinated and attuned conversation occurs when one person speaks while the other listens and vice versa; (2) Leader–follower dynamics, where one person temporarily guides the flow of conversation while the other follows; and (3) Asymmetries in nonverbal interactional dominance, which reflect how one partner may control pauses and speech timing more than the other. Detailed operationalizations of these dynamics and their measurements are provided in the Method section. We also examine how individual differences in Extraversion and Agreeableness impact these conversational dynamics.

#### Coordination in Social Interactions

Coordination can be understood as a self-organized set of coupled components that function as a single functional unit —such as a conversation (Bernieri et al., 1988; Shockley et al., 2009). These components are self-organized and context-sensitive, as each individual actively structures exchanges with their environment to create and sustain systemic stability (Varela et al., 2017; Thompson, 2007). Through this process, interacting partners develop stable, yet flexible patterns that are context-sensitive, self-organized, and adaptable (Thompson, 2007); allowing them to pursue opportunities (or affordances) aligned with their goals —whether those goals are affiliative, competitive, problem-solving, or others (Fusaroli et al., 2014; Kelso et al., 1984; Shockley et al., 2003). In essence, the actions of one partner influence the other, leading the dyad to behave as a coupled system (Shockley et al., 2009).

In our study, speech coordination refers to the interdependent dynamics of conversational elements, such as turns of speech and silence, that evolve together over time with a coherent rhythm. This coherence does not imply that participants are performing the same actions simultaneously. Rather, they engage in mutually dependent and adaptive dynamics, where each partner adjusts to the other, allowing specific functions and goals to emerge within the conversation (Nowak et al., 2017). We define speech coordination as a reciprocal, nonverbal process of turn-taking that creates a rhythmic flow in dialogue (Reuzel et al., 2013). Our analysis focuses on speech coordination, the temporal aspects of leader–follower dynamics, and the asymmetries in nonverbal interactional dominance that manifest during turn-taking (Reuzel et al., 2014).

#### Nonverbal Interactional Dominance in Speech and Personality

Speech coordination occurs when interacting partners establish an optimal, predictable rhythm in conversation, facilitating smooth turn-taking with fewer interruptions or extended silences (Warner, 1992; Reuzel et al., 2013). These coordinated rhythms are associated with affiliation (Hove & Risen, 2009), cooperative efficiency (Delaherche et al., 2012), successful negotiation outcomes (Di Stasi et al., 2024), and positive affect (Warner, 1992). However, speech distribution in conversation is not always equal. One partner may dominate the interaction, speaking more frequently and exerting greater influence, a behavior linked to higher status and decision-making power (Bales, 1973; Meeker, 2020). Conversation dominance can be indicated by the speed of speech onset, or response latency, as well as other nonverbal cues like *leader–follower* dynamics in turn-taking (Berger et al., 1972; Fişek et al., 2005; Meeker, 2020; Reuzel et al., 2013, 2014). Individuals who initiate speech more often and lead conversational dynamics can create asymmetries in the flow, where one person consistently drives the conversation by starting more speaking episodes (Genschow & Alves, 2020). Additionally, nonverbal interactional dominance refers to the degree and duration of these imbalances, where one partner's nonverbal behavior exerts greater *influence* over the other (Reuzel et al., 2014).

Conversation dynamics—such as speech coordination, leader–follower roles, and nonverbal dominance—allow individuals to meet their communicative goals. However, these dynamics are also influenced by situational factors and individual differences between partners (Mischel & Shoda, 1995). Thus, we investigate how variations in Extraversion and Agreeableness shape these interactional patterns.

#### Personality Conceptualizations and the Emergence of Dyadic Systems

Dynamic personality models propose that while human affect, behavior, thoughts, desires, and action predispositions continuously change due to intrinsic and external forces, they eventually converge into stable personality patterns over time (Bleidorn et al., 2022; Nowak et al., 2005; Revelle & Wilt, 2020; Sosnowska et al., 2019). Personality differences can be seen as tendencies to optimally engage with the world, reflecting individual variations in person-environment fit (Hovhannisyan & Vervaeke, 2022). Thus, personality represents how individuals navigate and structure social affordances across contexts (Chemero, 2003; Gibson, 1979; Satchell et al., 2021), offering a broader, dynamic perspective compared to traditional trait models—often termed characteristic adaptations (Nguyen et al., 2021).

Personality also describes how differences between conversation partners influence the emergence of higher-order conversational elements within the dyadic system (Mischel & Shoda, 1995; Nowak et al., 2020). In this study, we examine how the personality traits of each partner affect dyadic speech coordination across different types of conversations, and how the partners appraise these interactions. We focus on Extraversion and Agreeableness, two core dimensions of social behavior (Goldberg et al., 1998; Koole et al., 2001; McCrae & Costa, 2008), whereas the remaining three traits of the Five-Factor Model—Conscientiousness, Neuroticism, and Openness—are more relevant to specific domains such as work, emotional regulation, and intellectual pursuits (Cuperman & Ickes, 2009; Larsen et al., 2025; Peabody & Goldberg, 1989).

Extraversion reflects sociability, with higher scores being lively, outgoing, and adventurous, in contrast to introverts (McCrae & Costa, 2008). Agreeableness reflects tendencies toward altruism and cooperation, while disagreeable individuals often lack concern for others (DeYoung, 2015; Hovhannisyan & Vervaeke, 2022). Previous studies show that individual speech patterns are influenced by the talkativeness of the partner, partly reflecting their personality traits (Borgatta & Bales, 1953; Leaper & Ayres, 2007; Oben & Brône, 2016). For instance, concordant extraverted dyads tend to cover a wider range of topics and engage in more personal self-disclosure, while introverted dyads often focus on problem-solving themes and are more concise (Arellano-Véliz et al., 2024b; Cuperman & Ickes, 2009; Thorne, 1987). Additionally, dyads with higher levels of Extraversion exhibit greater nonverbal rhythmic synchrony, a finding consistently observed across video-tracking studies (Fujiwara & Yokomitsu, 2021). Concordant dyads, whether introverted or extroverted, often rate their interactions as more positive and engaging. In contrast, discordant dyads in Agreeableness (agreeable/disagreeable) may disclose more personal information, with agreeable individuals generally evaluating the interaction more positively (Arellano-Véliz et al., 2024b). Overall, the role of conversational topics and personality traits in shaping speech coordination remains an open question.

### The Present Study

We propose that speech coordination dynamics, as emergent properties of conversation, are shaped by a complex interplay of personality traits and contextual/conversational goals. To investigate these interpersonal dynamics, we analyzed time series of nonverbal conversational behavior, focusing on three key aspects: overall speech coordination, leader–follower dynamics, and asymmetries in nonverbal interactional dominance. We aimed to elucidate how high-level situational constraints, such as conversational topics (i.e., introductory, self-disclosure, and argumentative discussions), influence these dynamics (Paxton & Dale, 2017). Similarly, we explored how individual differences in Extraversion and Agreeableness modulate conversational patterns. Furthermore, we scrutinized how these factors shape interaction partners' perceptions and evaluations of the conversation.

#### Cross-Recurrence Quantification Analysis to Quantify Coordination of Speech, Leader–Follower Dynamics, and Nonverbal Interactional Dominance

To explore speech coordination, leader–follower dynamics, and asymmetries in nonverbal interactional dominance, we applied three nonlinear time-series techniques based on Cross-Recurrence Quantification Analysis (CRQA; Cox et al., 2016; Marwan et al., 2007; Zbilut & Webber, 1992), as outlined in the method section. These methods provide a robust framework for identifying temporal patterns and interdependencies in speech dynamics during interpersonal interactions (Cox et al., 2016; Marwan et al., 2007; Zbilut et al., 1998). Our analysis primarily focused on turn-taking behavior, particularly moments when one partner spoke while the other remained silent, akin to the approach of Reuzel et al., (2013, 2014).

The nonlinear time series approach enabled us to examine interpersonal speech dynamics across three domains. First, we assessed speech coordination *globally* (across all time lags) and *simultaneously* (at lag-zero), identifying instances where one partner’s silence aligned with the other’s speaking. Second, we evaluated leader–follower dynamics by quantifying the imbalance in turn-taking initiatives, providing insights into conversational balance. Third, we examined asymmetries in nonverbal interactional dominance by measuring how one partner’s behavior influenced the other.

These complex dynamic systems techniques are valuable for studying dyadic interaction dynamics and the influence of personality traits on reciprocal interactions (Mischel & Shoda, 1995). Individual differences can shape the dyadic system, leading to emergent properties that would not be present otherwise, such as how a talkative person might create more opportunities for interaction. Integrating CRQA with dyadic systems and personality traits offers a comprehensive framework for understanding the multifactorial nature of interpersonal dynamics.

#### Expectations

This study aimed to answer four research questions, with the corresponding hypotheses outlined in Table 1 (See method section for the operationalization of the variables; Table 2).^1^

**Table 1:** Expectations by each research question

Research question	Hypothesis
(1) How do conversational constraints influence speech coordination, leader–follower dynamics, and nonverbal interactional dominance?	*H1a:* Conversational type will significantly explain variance in speech coordination. Self-disclosing (Thorson et al., 2021) and argumentative conversations (Tschacher et al., 2018) will result in higher coordination, driven by affiliative or competitive motivations (Allsop et al., 2016)
	*H1b:* Conversational constraints will lead to differences in leader–follower dynamics and nonverbal interactional dominance, with stronger effects expected in argumentative tasks compared to introductory conversations (Paxton & Dale, 2017; Reuzel et al., 2014)
(2) How do personality traits and dyad composition influence speech coordination?	*H2a:* Higher Extraversion within dyads will increase speech coordination due to extraverts’ social enjoyment and talkativeness (Funder & Sneed, 1993; Leaper & Ayres, 2007; Cuperman & Ickes, 2009; Fujiwara & Yokomitsu, 2021)
	*H2b:* The presence of at least one extrovert in a dyad will increase coordination, as the more socially engaged partner drives alignment (e.g., Arellano-Véliz et al., 2024b; Lucas & Diener, 2001; Tuovinen et al., 2020)
	*H2c:* High Agreeableness will increase coordination due to warmth and friendliness (Funder & Sneed, 1993; Graziano et al., 2007; Graziano & Tobin, 2009; Cuperman & Ickes, 2009)
	*H2d:* Low Agreeableness will inhibit coordination, particularly in dyads with at least one disagreeable partner. Disagreeable individuals are less likely to engage in prosocial behaviors, such as active listening and responsiveness, which are essential for effective speech coordination and attunement (e.g., Graziano & Tobin, 2009; Graziano et al., 2007)
	*H2e:* An exception to H2d might occur during argumentative conversations, where low Agreeableness could lead to increased coordination (as evidenced in the case of body motion, Arellano-Véliz et al., 2024b). This may be due to competition motives (Urbig et al., 2021), however, this explanation is still exploratory
(3) How do personality traits and dyad composition influence leader–follower dynamics and nonverbal interactional dominance?	*H3a:* Higher Extraversion will correlate with more imbalanced leader–follower dynamics, where extroverts take the lead more often (Funder & Sneed, 1993; Leaper & Ayres, 2007; Cuperman & Ickes, 2009)
	*H3b:* High Agreeableness will foster balanced dynamics, while low Agreeableness will lead to greater speech disparities and dominance asymmetries (Arellano-Véliz et al., 2024b; Urbig et al., 2021)
(4) How do speech coordination, leader–follower dynamics, and nonverbal interactional dominance affect perceived interaction quality?	*H4a:* Higher coordination will positively influence perceived interaction quality, reflecting more positive and attuned interactions (Arellano-Véliz et al., 2024b; Graziano et al., 2007; Hove & Risen, 2009; Warner, 1992)
	*H4b:* Balanced leader–follower dynamics and symmetric interactions (i.e., lower dominance asymmetry) will contribute to more positive interaction perceptions (as evidenced by Reuzel et al., 2014)
	*H4c:* Higher Extraversion will correlate with more positive interaction perceptions due to more dynamic and engaging conversations (Funder & Sneed, 1993; Cuperman & Ickes, 2009)
	*H4d:* Higher Agreeableness will be linked to more positive perceptions, especially in attuned and cooperative contexts (Arellano-Véliz et al., 2024b; Warner, 1992)

## Method

### Participants

The data presented in this paper were collected between 2021 and 2022 as part of a larger multimodal experimental project (see [blinded] et al., 2024). From an initial pool of 300 screened participants, 112 undergraduate students participated in 15-min same-sex dyadic conversations conducted in a controlled laboratory setting. The final sample consisted of 100 participants (50 same-sex dyads) aged 18 to 33 years (*M* = 20.54, *SD* = 2.74; 72 females, 28 males), as only these data met the criteria for analysis due to the audio file integrity (six dyads were excluded for inadequate recordings). To control for potential confounding factors related to pre-existing relationships, participants were unacquainted before the study. All participants received ECTS credits for their involvement and provided informed consent in line with ethical standards for research involving human participants (ethical approval code PSY-1920-S-0525).

We initially designed the laboratory study to examine the impact of (dis)similarity in socially relevant trait scores, particularly Extraversion and Agreeableness, focusing on individuals scoring either 0.5 SD above (“high”) or 0.5 SD below (“low”) the sample mean (e.g., Li et al., 2020). However, in our models, we utilized the entire sample of 100 participants, modeling the personality traits continuously while maintaining the dyadic structure parsimoniously. Additionally, we provide descriptive statistics and plots following this threshold-based dyadic matching, similar to the approach outlined by Li et al. (2020) for low/high trait scores and by Cuperman and Ickes (2009). To simplify the interpretation of the results, the participant with the higher score within the dyad is designated as participant "A," while the one with the lower score is designated as participant "B.

### Instruments

#### Big Five Personality Traits: International Personality Inventory Pool—120 (IPIP-NEO-120)

Personality traits were assessed using the IPIP-NEO-120 (Johnson, 2014) via the Qualtrics online platform approximately ten days before the laboratory study. Participants provided informed consent before completing the self-report questionnaire, which measures the Big Five personality traits—Extraversion, Neuroticism, Openness to Experience, Agreeableness, and Conscientiousness—along with their 30 facets. The questionnaire consists of 120 items and typically takes 10 to 20 min to complete (Johnson, 2014). The IPIP-NEO-120 has demonstrated psychometric properties consistent with the NEO-PI-R scales (McCrae & Costa, 2008), indicating its reliability and validity. In a sample of 501 individuals, the IPIP-NEO-120 showed high correlations with the NEO-PI-R across all five traits (Extraversion 0.85, Neuroticism 0.87, Openness 0.84, Agreeableness 0.76, and Conscientiousness 0.80; all *p* < .01; Johnson, 2014). The questionnaire also exhibited good internal consistency, with Cronbach's alpha values of 0.84 to 0.88 across the five traits. The IPIP-NEO-120 is publicly available, cross-culturally robust, and suitable for use with international samples.

#### Self-disclosure Paradigm

We used the self-disclosure paradigm to promote affiliative conversation (Aron et al., 1997). This protocol involves partners asking and answering progressively personal questions to foster interpersonal closeness. The original version consists of three sections with 12 questions each, taking about 45 min to complete. For our study, we shortened the protocol to three sets of three questions (9 questions total) to fit within a 5-min conversation segment. Participants were asked to select at least one question from each set, with both partners answering each question and sharing as much as they felt comfortable. Sample questions include:* 'What would constitute a perfect day for you?', 'Is there something you’ve dreamed of doing for a long time? Why haven’t you done it?', and 'How do you feel about your relationship with your family?'".*

#### Perception of the Interaction (Appraisals)

After the dyadic conversation, participants were asked to complete a modified version of the Perception of the Interaction questionnaire (Cuperman & Ickes, 2009) to assess the self-reported interaction experience. The scores go from 1 (“not at all”) to 5 (“very much”). The original version of this questionnaire assesses the first-person perspective (e.g., *“To what extent did you feel accepted and respected by the other person”*?) and the third-person perspective (e.g., *“To what extent do you think your conversation partner felt accepted and respected by you?*). In the present study, we report the first-person questions, as our research focused on individual experience, instead of actor-partner interdependence effects. To preserve nuanced interpretations the Perception of Interaction questionnaire *items* were used as variables, instead of clusters of items, following the precedent by Funder and Sneed (1993) and Cuperman and Ickes (2009).

### Procedure

Participants were invited to the experimental study and provided with a heart rate transmitter belt upon arrival at the laboratory, though these data are not included in this paper. After arriving, participants read and signed the informed consent form and completed an affect questionnaire (PANAS, 2007; not reported in this paper, but consult [blinded] et al., 2024). A microphone was then attached to their clothing, and they received instructions about the conversation task. The interaction took place with participants standing face-to-face on a balance board (designed to measure postural control, not reported here) at a fixed distance of 1.5 m. The balance board also served as a convenient positioning device to keep people at a fixed distance and facing each other. A camera positioned about 4.5 m away recorded the interaction from a sagittal perspective.

The conversation followed a semi-structured schedule lasting approximately 15 min, divided into three 5-min parts, though participants could move to the next phase at their discretion. The interaction included (1) introductions, (2) self-disclosure, and (3) an argumentative conversation or debate. In the introduction phase, participants briefly introduced themselves, with general themes provided for guidance if needed. During the self-disclosure conversation, participants followed a shorter version of the self-disclosure paradigm, as detailed in the previous section. In the argumentative phase, participants selected a topic from a pool of around 20 and took opposing sides (pro/against) on issues like "Are strict lockdowns a valid measure during the pandemic to keep people safe?", "Are dating apps a good platform for meeting a romantic partner?", and "Should pre-adolescents and adolescents use social media freely?". They debated as many topics as possible within 5 min, with time monitored by an alarm, but could extend the discussion as needed before moving to the next phase. After the interaction, participants completed questionnaires on affective state, interpersonal closeness, and interaction appraisals.

### Data Processing and Time Series Generation

**Fig. 1:**
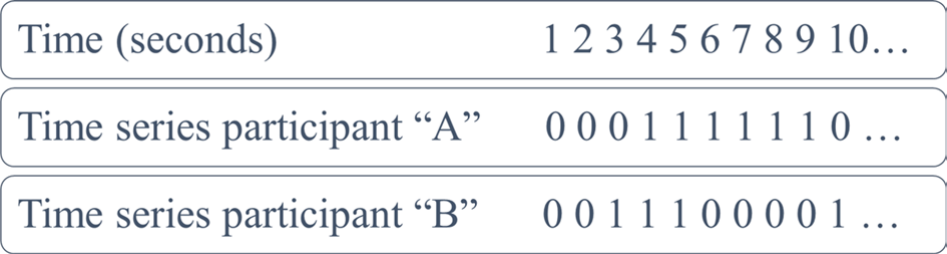
Representation of the time series generation. *Note*: The figure represents the coding of the time series, which represents the speech (1) and silence (0) segments by seconds in the time series of each interacting partner (Figure adapted from Reuzel et al., 2013)

Data streams were recorded using Lab Streaming Layer software (Kothe et al., 2019). Each audio stream was cleaned with Adobe Audition, utilizing the default ‘noise print’ and ‘DeNoise’ functions to minimize background noise and enhance the clarity of participants' voices. The cleaned files were then analyzed using the voice activity annotation function in Praat software (Boersma & Weenink, 2023), which coded silence and speech segments as ‘0 = silence’ and ‘1 = speech’, following the procedure outlined by Reuzel et al. (2014). This process produced a dichotomous time series of 1’s and 0’s for each interacting partner (see Fig. 1). To ensure sensitivity to phonetic details, we resampled the time series to align with utterance levels, defining each silence and speech segment as 1 s in duration (e.g., as per the Situation Model by Abney et al., 2014; Pickering & Garrod, 2004).

### Time Series Technique and Statistical Analyses

#### Categorical Cross-Recurrence Quantification Analysis (CRQA)

**Fig. 2:**
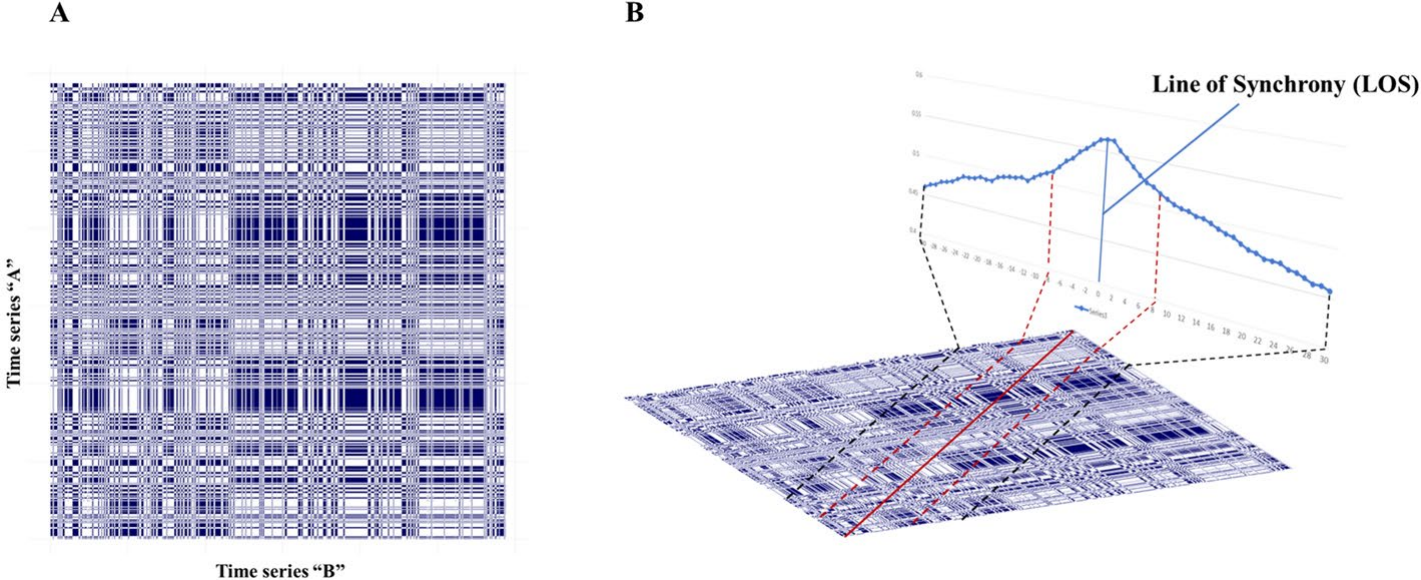
Cross-recurrence plot and diagonal cross-recurrence profile. *Note*: **A** The figure represents a categorical cross-recurrence plot. In this case, the matching between the two interacting partners (time series) was defined as one person speaking (categorized as 1) and the other person being silent (categorized as 0). In the plot, these occurrences are represented by the blue lines or blocks, whereas the non-occurrences —both speaking (1–1) or both in silence (0–0)— are represented by white spaces. **B** Diagonal Cross-Recurrence Plot (DCRP) representation. Adapted from Wallot and Leonardi (2018). The profile can help determine the coupling direction of time series in terms of leading and following dynamics at different lags along the line of synchrony (LOS). Each line parallel to the line of synchrony represents a particular delay or lag in the alignment of speech dynamics between both interacting partners. A lag of 0 indicates synchrony or simultaneous recurrence. In the context of turn-taking, this represents moments when both individuals are engaged in speaking or listening at the same time, suggesting coupling, reciprocity, and attunement.

We employed a categorical *Cross-Recurrence Quantification Analysis (CRQA)* to measure speech coordination or dyadic coupling. This nonlinear bivariate correlation technique quantifies the temporal similarity or coupling properties between time series, specifically focusing on conversation partners (Marwan et al., 2007; Wallot & Leonardi, 2018; Zbilut et al., 1998). The core of CRQA involves constructing a cross-recurrence plot, which visually represents instances where the behaviors of the two time-series match (Cox et al., 2016; Xu et al., 2020; Wallot & Leonardi, 2018; see Fig. 2). These recurrence measures elucidate the temporal dynamics of the interacting systems across various lags or time scales (Marwan et al., 2007; Zbilut et al., 1998).

Given the categorical (dichotomous) nature of our data, we utilized a categorical form of CRQA. Each interaction partner's behavior was coded as “1” for speech and “0” for silence (see Fig. 1). A match (recurrence) is recorded as a dot in the cross-recurrence plot when one partner speaks while the other is silent (i.e., combinations of “1” and “0” or “0” and “1”). Thus, a line in the plot represents the prolonged co-occurrence of speech and silence, allowing us to quantify the coordination or attunement between partners during the conversation (Reuzel et al., 2013). Recurrences in dyadic behavior can occur simultaneously or in temporal proximity, reflecting some delay (Cox et al., 2016).

Categorical CRQA has been successfully applied across various domains to study the coupling between two time-series (Coco & Dale, 2014; Cox et al., 2016; Wallot & Leonardi, 2018). It has identified rhythmic structures in complex human behavior, such as speech and body motion (Arellano-Véliz et al., 2024a; Kodama et al., 2021). In interpersonal coordination, CRQA robustly identifies dynamics using categorical data in diverse contexts, including client-therapist interactions (Reuzel et al., 2013, 2014), mother-infant dynamics (Lira-Palma et al., 2018), and various modalities of ambulatory social behavior (Danvers et al., 2020).

We conducted two follow-up analyses: *Diagonal Cross-Recurrence Profile (DCRP)* and *anisotropic CRQA (aCRQA)*. DCRP analyzed leader–follower dynamics by quantifying the imbalance in turn-taking initiatives between partners. aCRQA examined differences in nonverbal interactional dominance, measuring the extent and average duration of asymmetric episodes influenced by one partner’s nonverbal behavior on the other. Definitions for all variables in this study, including *RR*_*global*_ (recurrence rate across the entire cross-recurrence plot) and *RR*_*LOS*_ (recurrence rate at lag zero), are provided in Table 2.

**Table 2:** Cross-recurrence quantification analysis: measures, description, and interpretations

Measure	Formula and description	Interpretation
*Overall Speech Coordination (coupling)—CRQA/DCRP*		
*Recurrence Rate* *RR*_*global*_	*Sum of recurrent points in cross-recurrence plot *$\div$* the size of cross-recurrence plot* Measures the overall rate (proportion) of recurrent points (matches) between the two time series across the entire cross-recurrence plot. Matching was specified as one person speaking while the other was silent (1–0, 0–1 occurrences).^11^	*RR*_*global*_ estimates coordination across all possible lags (delays). Higher *RR*_*global*_ indicates more speech coordination across all possible lags (greater engagement in turn-taking episodes), and more structured and responsive interactions. A low(er) *RR*_*global*_ indicates less coordination, more interruptions, silences, and a more irregular pattern of coupling.^1^ This global measure of coordination captures long-range matches or long-range coordination patterns that are common in complex systems’ behavior
*Recurrence Rate through the Line of Synchrony* *RR*_*LOS*_	*Sum of recurrent points on the line of synchrony ÷ length of the line of synchrony(length of the time series)* The rate (proportion) of recurrence (matching) on the line of synchrony represents the instances where speech and silence co-occur (simultaneously). It represents the percentage of synchrony.^1,2,3^	*RR*_*LOS*_ is a measure of synchrony or coupling in speech at lag-zero (simultaneous). Higher *RR*_*LOS*_ suggests more co-occurrence of speech and silence thus coordination at the same time and, therefore, responsive interactions. Conversely, a lower *RR*_*LOS*_ indicates less coordination, more interruptions, and silences at lag-zero (simultaneously).^1^
*Leading-Following Dynamics (DCRP)*		
*Quotient of Diagonal Cross Recurrence Profile,* *Q*_*DCRP*_* (absolute)*	*|(RR*_*right*_*—RR*_*left*_* ÷ RR*_*right*_ + *RR*_*left*_*)|* *RR*_*right*_ and *RR*_*left*_ refer to the recurrence rates on the left and right sides of the LOS, respectively, within the DCRP (see Figs. 2B and 6).^1, 2, 3^	*Q*_*DCRP*_ indicates the absolute overall degree of balance/imbalance between the left and right sides along the LOS.^4^ Leading-follower dynamics can be inferred from this measure, where 0 represents a completely balanced interaction (i.e. equally leading and following), and 1 represents the situation where one of the interacting partners is leading during the entire interaction.^1^
*Asymmetries in Nonverbal Interactional Dominance (aCRQA)*		
*Relative Difference in Laminarity of Anisotropic CRQA (absolute),* *LAM*_*ARD*_	*|(LAM*_*ver*_*—LAM*_*hor*_*)|÷ (LAM*_*ver*_ + *LAM*_*hor*_*)|* *LAM*_*ver*,_ and *LAM*_*hor*_ refer to the Laminarity of vertical (interacting partner “A”) and horizontal lines (interacting partner”B”), respectively. Laminarity is a recurrence measure that quantifies the presence of vertical and horizontal lines in the *DCRP*, indicating periods of repeated fixed behavioral patterns in the dynamics of the conversation. The absolute relative difference in laminarity compares the laminarity along specific directions (i.e. horizontal and vertical), reflecting variations in the leading-following (balance) dynamics between conversational partners.^4^	Asymmetric laminar patterns indicate conversation dominance. A higher relative difference in laminarity suggests the overall asymmetry's magnitude in the interactions' nonverbal interactional dominance. If one dyadic partner more often initiates the talking and silences this steers episodes of silence or speech in the other. Hence, one person “affords” or gives the other opportunities to display behavior to the partner, and thus is more “dominant” or influential in the conversation.^4^ Low *LAM*_*ARD*_ implies a more “balanced” interaction when nobody consistently takes the lead over the other The absolute value of the relative difference captures the *size* of the difference within this measure between the members of each dyadic system
*Relative Difference in Trapping Time of Anisotropic CRQA (absloute),* *TT*_*ARD*_	*|(TT*_*ver*_*—TT*_*hor*_*)|*/*(TT*_*ver*_ + *TT*_*hor*_*)|* *TT*_*ver*_ and *TT*_*hor*_ refer to the Trapping Time of vertical (interacting partner “A”) and horizontal lines (interacting partner”B”) respectively. Trapping Time quantifies the average duration for which one system "traps" or captures the behavior of the other system. It quantifies the average temporal persistence of one system's influence on the other.^4^	One partner's speech and silence episodes lead ("trap") the other partner in relatively *longer* or *shorter* episodes of silence and speech, respectively. A longer trapping time indicates a sustained influence of one speaker on the other or nonverbal interactional dominance. A shorter trapping time suggests swift reciprocal interactions, where both speakers influence one another. It can be indicative of a higher interactional *sensitivity* or *responsivity*.^4^ The absolute value of the relative difference captures the *size* of the difference within this measure between the members of each dyadic system

*CR plot* Cross-Recurrence plot, *RR* Recurrence Rate, *CRQA* Cross-recurrence quantification analysis. *aCRQA* Anisotropic Cross-recurrence quantification analysis. *DCRP* Diagonal Cross Recurrence Profile. *LAM* Laminarity. *LOS* Line of Synchrony. *TT* Trapping Time

References:^1^ Reuzel et al. (2014); ^2^ Richardson and Dale (2005), ^3^ Wallot and Leonardi (2018), ^4^ Cox et al. (2016). CRQA analyses were performed using Marwan’s CRP toolbox (2024, available at https://tocsy.pik-potsdam.de/CRPtoolbox) on MATLAB (2022)

To address concerns regarding distant 1–0 or 0–1 matches potentially reflecting chance coordination—especially when using global measures like *RR*_*global*_—we compared randomized (shuffled) data to real data, following the pseudo synchrony paradigm (Bernieri & Rosenthal, 1991). Coordination typically decreases with lag, a trend observed in body motion synchronization studies (Tschacher et al., 2018). This decline can be assessed using tools like DCRP. Validating these techniques is crucial to confirm that speech coordination, including turn-taking at both zero and global lags, differs from random occurrences. Incorporating a global measure of coordination (*RR*_*global*_) allows for capturing long-range memory influences in complex systems. Importantly, a decline in coordination at certain lags does not indicate a lack of memory; instead, distant matches may emerge from long-range dependencies, reflecting underlying interpersonal dynamics (see Chen et al., 2003; Marmelat & Delignières, 2011, for discussions on long-range dependencies and complexity in coordination).

#### Diagonal Cross-Recurrence Profile (DCRP)

We utilized *Diagonal Cross-Recurrence Profile analyses (DCRP)* to assess the balance and imbalance in leader–follower dynamics during dyadic conversations (Richardson & Dale, 2005). DCRP quantifies the number of recurrences at various lags along the main diagonal, or Line of Synchrony (LOS), of the cross-recurrence plot (see Fig. 2B; Wallot & Leonardi, 2018; Tomashin et al., 2022). The LOS captures instances of simultaneous matching behaviors at lag-zero, specifically when one participant speaks (code “1”) while the other is silent (code “0”). The diagonals parallel to the LOS display instances where these recurrences (matching behaviors) occur with some delay, which increases the further one moves away from the LOS. The distribution of recurrences at one side of the LOS indicates how much (and how quickly) the behaviors of one participant in the time series are followed by the matching behaviors in the other participant for different lags. Importantly, it is likely that the recurrences on both sides of the LOS are asymmetrically distributed (Wallot & Leonardi, 2018), creating a diagonal cross-recurrence profile (DCRP). The DCRP quantifies leader–follower imbalances in the conversation (Dale et al., 2011; López Pérez et al., 2017). We computed the absolute Quotient of the DCRP (*Q*_*DCRP*_) to indicate the overall conversational imbalance in leading and following between the interaction partners (see Table 2; Richardson & Dale, 2005; Dale et al., 2011).

#### Anisotropic CRQA (aCRQA)

To further investigate asymmetries in nonverbal interactional dominance during conversations, we employed *Anisotropic Cross-Recurrence Quantification Analysis* (*aCRQA*; Cox et al., 2016; Xu et al., 2020). Categorical time series typically form rectangular structures in cross-recurrence plots, indicating coupling between the two partners (see Fig. 2A). aCRQA quantifies the relative influence of each interacting partner by capturing matched behaviors over time, separately assessing the distribution of vertical and horizontal structures in the cross-recurrence plot (Cox et al., 2016; Xu et al., 2020). These structures represent behaviors from one partner that are matched by the other over extended periods, indicating how one partner may dominate or lead the interaction. The distinction between horizontal and vertical structures reveals the extent to which one interaction partner influences the other. aCRQA analyzes these lines separately and quantifies their relative differences in terms of quantity and length, with variations indicating asymmetries in dominance—specifically, unequal coupling strength between the interacting partners (Cox et al., 2016; López Pérez et al., 2017).

To quantify overall conversational asymmetries, we focused on two measures: Relative differences in Laminarity (*LAM*_*ARD*_) and Trapping Time (*TT*_*ARD*_) (see Table 2). Asymmetries in nonverbal interactional dominance indicate that one of the interaction partners tends to exhibit more control, *capturing* the other partner into matching behaviors for extended periods (van Dijk et al., 2018). This is not necessarily a negative aspect of the interaction, as the term ‘dominance’ might suggest. In this context, dominance describes potential asymmetries in the coupling strength of the dyadic system. We specifically examined turn-taking dynamics—episodes of speech and silence—represented by horizontal and vertical rectangular structures in the cross-recurrence plot. These structures illustrate how each partner creates opportunities for interaction. Thus, nonverbal interactional dominance, as reflected in these patterns, can positively enhance social communication by facilitating conversational flow.

We used absolute values for *Q*_*DCRP*_, *LAM*_*ARD*_, and *TT*_*ARD*_ to represent the *size* of the behavioral differences for each dyad. These values, derived from Diagonal Cross Recurrence Profiles and Anisotropic CRQA, provide a single measure per dyad, reflecting the dynamics at the dyadic level. This approach allows us to pair the dyadic measure with the personality traits of each partner. However, it is important to note that we can only assess how the personality traits of both partners affect the overall dyadic measure.

#### Models

We performed maximum likelihood mixed-effects models with two levels to test our research questions regarding interpersonal coordination of speech, task effects, and personality traits. Level 1 was the task or type of conversation (3 observations), nested within the dyadic structure (Level 2). We employed the "lme4" package in R, using the Satterthwaite method for calculating degrees of freedom and significance, as it is particularly suited for small sample sizes and complex model structures (Bates et al., 2015). First, we explored the effect of task on various outcomes,^2^ including speech coordination, leader–follower dynamics, and nonverbal interactional dominance. The predictor variable was the task (conversational topic), treated as a categorical variable with three levels (introduction, self-disclosure, and argumentative), with the introduction serving as the baseline. The response variables were *RR*_*global*_*, RR*_*LOS*_*, Q*_*DCRP*_, *LAM*_*ARD*,_ and *TT*_*ARD*_*,* respectively. Next, we modeled the effects of the personality traits of Extraversion and Agreeableness alongside the tasks on the same response variables.^3^ We reported both estimates and standardized beta weights (β) which can be interpreted as effect sizes (e.g., Paxton & Dale, 2013). For linear mixed effects, all continuous predictors were standardized before being incorporated into the models to obtain beta weights. Particularly, personality variables were centered by subtracting the mean and scaled by the standard deviation (R core team, 2022).

Subsequently, we investigated the impact of speech coordination and nonverbal interactional dominance on participants' perceptions of the interactions (appraisals assessment) using general linear models. We selected as predictors one variable of speech coordination (*RR*_*LOS*_) as this is the basic measure that provides information on speech coordination simultaneously (lag-zero); a variable informing about leader–follower dynamics from the DCRP analysis (*Q*_*DCRP*_), and one about nonverbal interactional dominance extracted from aCRQA (*LAM*_*ARD*_), which provides information about the extent of asymmetries in nonverbal interactional dominance. The items from the perception of interaction questionnaire served as the response variables, while the predictors included speech coordination, interactional dominance variables, and personality traits.^4^ To address potential Type I errors due to multiple hypothesis testing, we employed the Benjamini–Hochberg (BH) method for *p*-value correction (Benjamini & Hochberg, 1995).^5^

## Results

Descriptive statistics for all variables are provided in Table 3. Average speech time in seconds was the longest when participants introduced themselves (*mean* = 467.14, *SD* = 78.53), followed by self-disclosure (*mean* = 456.4, *SD* = 84.04), and shortest during the argumentative conversation (*mean* = 445.92, *SD* = 86.94).

**Table 3:** Descriptive statistics

Variable	1. Introduction				2. Self-disclosure				3. Argumentative			
	M	SD	Mdn	Range	M	SD	Mdn	Range	M	SD	Mdn	Range
Speech time (seconds)	467.14	78.53	461	[306, 616]	456.4	84.04	469	[258, 617]	445.92	86.94	443.5	[230, 671]
*RR*_*global*_	0.46	0.06	0.47	[0.26, 0.54]	0.46	0.05	0.47	[0.32, 0.55]	0.47	0.06	0.49	[0.30, 0.58]
*RR*_*LOS*_	0.56	0.14	0.59	[0.17, 0.85]	0.55	0.14	0.56	[0.13, 0.80]	0.58	0.15	0.58	[0.10, 0.82]
*Q*_*DCRP*_	0.02	0.02	0.02	[0, 0.05]	0.03	0.02	0.02	[0, 0.12]	0.03	0.03	0.03	[0, 0.10]
*LAMa*	0.93	0.03	0.94	[0.82, 0.98]	0.94	0.04	0.95	[0.81, 0.99]	0.94	0.04	0.95	[0.75, 0.99]
*TTa*	8.17	2.63	7.92	[4.24, 18.8]	8.74	4.46	8.07	[3.7, 46.29]	9.51	3.77	8.7	[4.55, 25.95]
*LAM*_*ARD*_	0.02	0.02	0.02	[0, 0.11]	0.02	0.02	0.02	[0, 0.07]	0.02	0.02	0.02	[0, 0.07]
*TT*_*ARD*_	0.15	0.13	0.09	[0.01, 0.54]	0.16	0.13	0.12	[0.11, 0.53]	0.16	0.10	0.13	[0.01, 0.44]

N = 100 participants (50 dyads). *M* = mean, *SD* = standard deviation, *Mdn* = median. *RR*_*global*_ = Recurrence Rate Global, *RR*_*LOS*_ = Percentage of Speech across the line of Synchrony (lag-zero), *Q*_*DCRP*_ = Quotient Diagonal Cross-Recurrence Profile (absolute), *LAMa* = anisotropic Laminarity, *TTa* = *anisotropic Trapping Time, LAM*_*ARD*_ = Relative difference of anisotropic Laminarity (absolute), *TT*_*ARD*_ = Relative difference of anisotropic Trapping Time (absolute). Anisotropic Laminarity (*LAMa)* and anisotropic Trapping Time (*TTa*) are the measures from which the relative difference of anisotropic Laminarity (*LAM*_*ARD*_) and Trapping Time (*TT*_*ARD*_) were calculated respectively

**Fig. 3:**
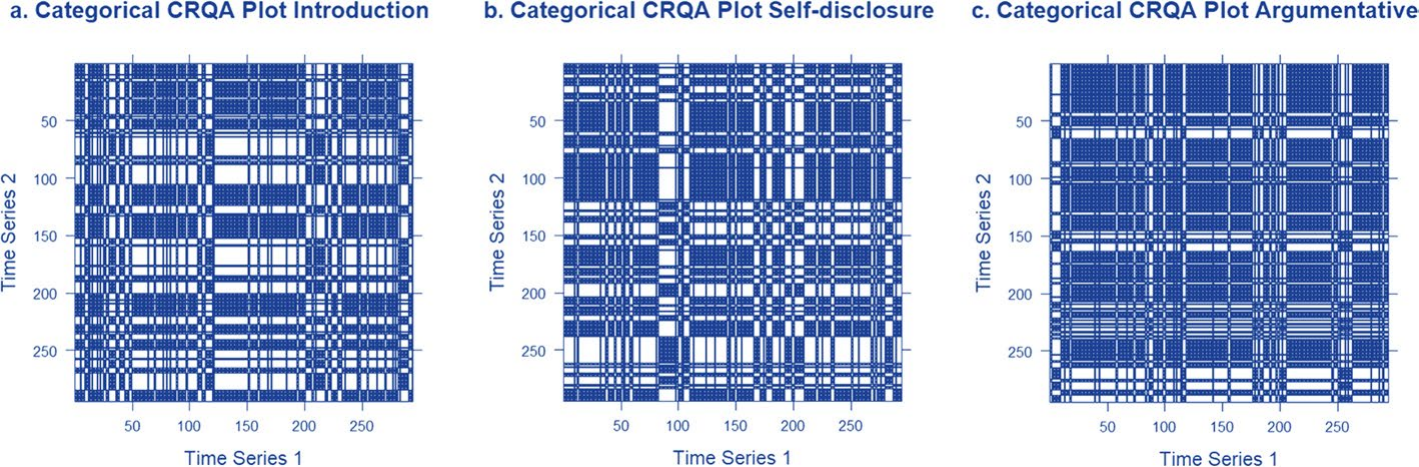
Cross-recurrence plots.* Note*: Cross-recurrence plots depict interaction dynamics in three tasks: **A** Introduction, **B** Self-disclosure, **C** Argument. The horizontal and vertical axes, “Time Series 1” and “Time Series 2” respectively, represent the time series of both interacting partners. Vertical lines represent temporal influence from one partner to the other, while horizontal lines signify reciprocal influence. Dark lines indicate matching behavior (speaking/silent); and white spaces indicate non-matching behaviors (e.g., simultaneous talking or silence). In the introduction (**A**), scattered patterns suggest exploratory interaction, with instances of one participant leading. Self-disclosure (**B**) shows pronounced matching blocks, indicating one participant's stronger influence. In the argumentative task (**C**), behaviors are evenly distributed, reflecting mutual temporal influence and response between participants

**Fig. 4:**
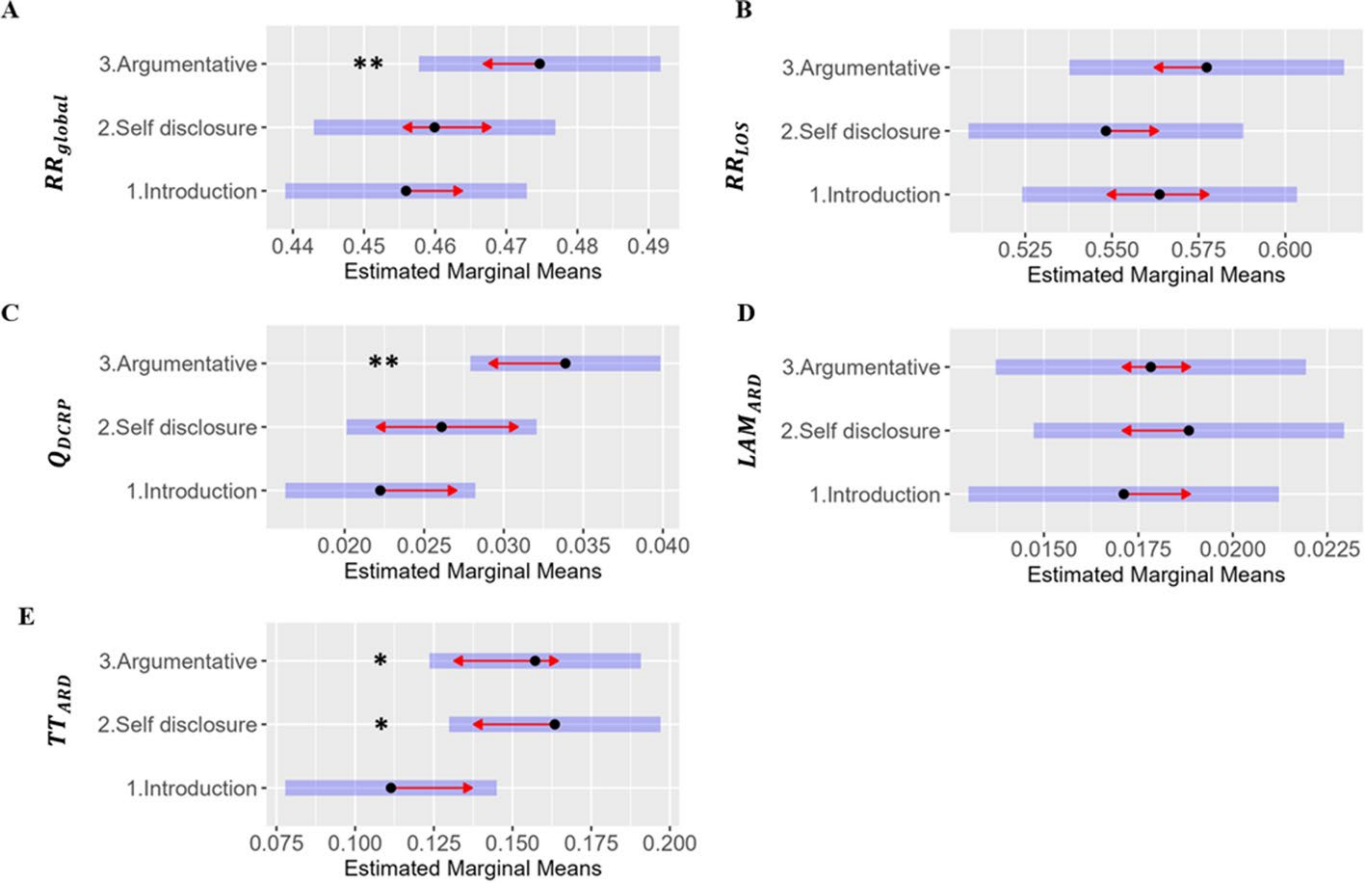
Estimated Marginal Means of topic predicting each CRQA, DCRP, and aCRQA measure. *Note*: * *p* < .05, ** *p* < .01. The plots represent the estimated marginal means for each measure of speech dynamic organization. The central points or markers represent the adjusted means of the response variable for different levels of the predictor variables, accounting for the effects of other variables in the model. The blue bars are confidence intervals for the Estimated Marginal Means, and the red arrows indicate comparisons between the means of the tasks with the baseline task (introduction). *CRQA* = Cross-Recurrence Quantification Analysis, *DCRP* = Diagonal Cross Recurrence Profile. *aCRQA* = anisotropic Cross-Recurrence Quantification Analysis. *RR*_*global*_ = Global recurrence rate (speech coordination at all lags). *RR*_*LOS*_ = Recurrence rate across the line of synchrony (lag-zero). *Q*_*DCRP*_ = Quotient of Diagonal Cross Recurrence Profile (balance in leader–follower dynamics). *LAM*_*ARD*_ = Laminarity absolute relative difference (asymmetries in nonverbal interactional dominance). *TT*_*ARD*_ = Trapping Time absolute relative difference (duration of episodes of nonverbal interactional dominance)

Using mixed-effects models we assessed whether dyadic speech coordination, leader–follower dynamics, and nonverbal interactional dominance differed across the three conversation topics. The Introduction task served as the baseline and we examined response variables including the Recurrence Rate Global (*RR*_*global*_), Percentage of Speech across the Line of Synchrony (lag-zero) (*RR*_*LOS*_*)*, Quotient Diagonal Cross-Recurrence Profile (absolute) (*Q*_*DCRP*_), Relative difference of anisotropic Laminarity (*LAM*_*ARD*_), and Relative difference of anisotropic Trapping Time (*TT*_*ARD*_; descriptives are provided in Table 3). Cross-recurrence plots of dyads during the three tasks are illustrated with one example in Fig. 3. Mixed-effect models estimates are provided in Fig. 4 and Table 4.

**Table 4:** Mixed-effects models predicting CRQA, DCRP and aCRQA measures from tasks with 50 dyads (N_i_) (100 participants) and 150 observations (N_t_), (50_i_ * 3 tasks)

Predictors	M1. *RR*_*global*_		M2. *RR*_*LOS*_		M3. *Q*_*DCRP*_		M4. *LAM*_*ARD*_		M5. *TT*_*ARD*_	
	Estimate B (*SE*)	*t*	Estimate B (*SE*)	*t*	Estimate B (*SE*)	*t*	Estimate B (*SE*)	*t*	Estimate B (*SE*)	*t*
Intercept	0.456 (0.008)	53.99***	0.564 (0.021)	28.69***	0.022 (0.002)	7.43***	0.017 (0.002)	8.32***	0.111 (0.017)	6.62***
Task 2	0.004 (0.007)	0.61	− 0.015 (0.013)	− 1.23	0.004 (0.003)	0.97	0.002 (0.002)	0.69	0.052 (0.021)	2.43*
Task 3	0.019 (0.007)	2.86**	0.014 (0.126)	1.08	0.011 (0.004)	2.92**	0.001 (0.002)	0.29	0.046 (0.021)	2.14*
*Random effects*										
ICC	0.70		0.80		0.70		0.27		0.19	
Marg. R^2^/ Cond. R^2^	0.02/0.70		0.01/0.80		0.01/0.70		0.002/0.27		0.04/0.22	
AIC	− 461		− 247		− 693		− 811		− 189	

Significance was indicated in bold: *p < .05. ^**^ *p* < .01,^***^p < .001. N_i_ = number of participants. N_t_ = total; number of observations, which was = 150 (50 dyads * 3 tasks). SE = Standard Error. Task 2 = Self-disclosure. Task 3 = Argument. Task 1 (Introduction) was considered the baseline in the models. AIC = Akaike’s Information Criterion (lower values indicate better fit). ICC = Intra-class Correlation Coefficient. See Table 2 for definitions. *RR*_*global*_ = Recurrence Rate Global, *RR*_*LOS*_ = Percentage of Speech across the line of Synchrony (lag-zero), *Q*_*DCRP*_ = Quotient Diagonal Cross-Recurrence Profile (absolute), *LAMa* = anisotropic Laminarity, *TTa* = *anisotropic Trapping Time, LAM*_*ARD*_ = Relative difference of anisotropic Laminarity, *TT*_*ARD*_ = Relative difference of anisotropic Trapping Time. Definitions of each measure can be found in Table 2

### Interpersonal Speech Dynamics of Speech by Conversation Topic

Our global measure of speech coordination (*RR*_*global*_) differed between conversational topics (see Fig. 4A), being lowest during the introduction and highest during the argumentative conversation (argumentative > introduction, *t*_(100)_ = 2.86, *p* < .01), which aligns with H1a, and indicates higher coordination of turn-taking behaviors across all lags. Similarly, imbalances in leading-follower dynamics (*Q*_*DCRP*_) were higher during the argumentative conversation than during introductions (argumentative > introduction, *t*_(100)_ = 2.92, *p* < .01), in line with H1b (Fig. 4C). This indicates imbalances between the interaction partners' initiative in turn-taking: one of the participants initiated or led more during dyadic conversations than the other who followed more (see Table 4).

During self-disclosure conversations the dyads showed greater asymmetries in the average duration of nonverbal interactional dominance episodes (or “trapping” episodes) than during introductions (*TT*_*ARD*_ self-disclosure > introduction, *t*(100) = 2.43, *p* < .05). Similarly, during the self-disclosure and argumentative conversations dyads showed longer “trapping” episodes (higher *TT*_*ARD*_, *t*(100) = 2.14, *p* < .05), in line with H2c (see Fig. 4E). Specific task effects were not significant for *RR*_*LOS*_ and *LAM*_*ARD*_*,* suggesting that speech coordination at lag-zero and the overall asymmetries of nonverbal interactional dominance did not significantly differ across tasks in our sample (see Figs. 4B and D, respectively).

The findings highlight that different conversational contexts—introduction, self-disclosure, and argumentation—impact speech coordination, leader–follower dynamics, and the duration of nonverbal interactional dominance. Argumentative conversations exhibited increased speech coordination and greater imbalances in leader–follower dynamics, while self-disclosure and argumentative interactions led to longer durations of nonverbal interactional dominance compared to introductions.

**Fig. 5:**
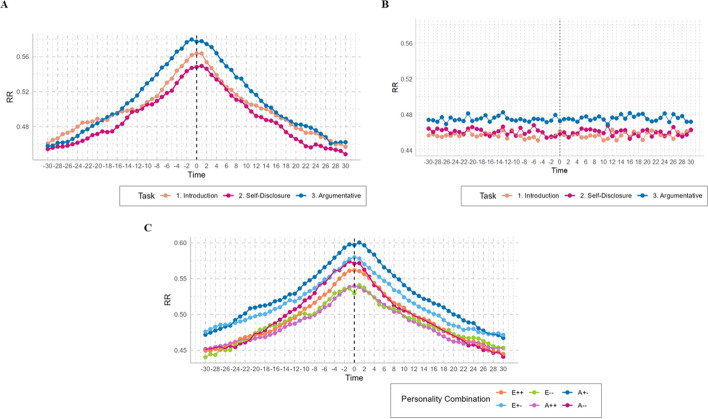
DCRPs by topic and personality combination. *Note*: The plots represent the DCRPs of speech coordination (turn-taking match) by topic with real data (**A**), the profiles with shuffled/randomized data (**B**), and real profiles by personality combination (**C**). The zero on the “x” axis (dashed line) represents the line of synchrony (LOS) corresponding to lag-zero. The “y” axis indicates the *RR* (percentage of recurrence rate), representing speech synchrony (or coupling) between interacting partners during the complete 15-min conversation. Lags to the sides of the line of synchrony line suggest that one behavior (i.e., speaking) is leading, and the other behavior (i.e., listening) is following after a certain time delay. This indicates a temporal pattern where one person initiates a turn, and the other responds after a specific duration (in seconds). In descriptive terms, in **A**, task 3 exhibits the highest *RR*, and task 2, the lowest. **B** (randomized data), there is no evidence of coordination in the line of synchrony (lag-zero). **C** the dyads composed of a person high and low in Agreeableness represent the highest *RR* and therefore, the strongest coupling, while the lowest RR is visualized in the dyads composed of two individuals with low scores on Extraversion. **B** regarding dyadic personality combinations: “E++” and “A++” = high/high scores; “E--” and “A--" = low/low scores; “E+-” and “A+-” = low/high scores. To plot the different dyadic compositions, thresholds of 0.5 *SD* were applied for high/low (e.g., Li et al., 2020) and only for visualization purposes

To assess the difference between real and random recurrences, we shuffled the time series while preserving the dyadic structure. We then averaged the DCRPs and compared the recurrences for each conversation topic (see Fig. 5A for real data and 5B for shuffled data). This allows for visual inspection, confirming that observed coordination effects, especially at lag-zero disappear when the time series are shuffled. T-tests comparing real data in the global measure of coordination at all lags (*RR*_*global*_) to chance revealed significant differences for all three conversation types. For the Introduction phase (*t* = −5.12, *p* < .001, 95% CI [0.44, 0.47]), Self-Disclosure (*t* = −5.17, *p* < .001, 95% CI [0.44, 0.48]), and Argumentative phase (*t* = −2.76, *p* = .008, 95% CI [0.46, 0.49]).

### Speech Coordination and Personality

**Fig. 6:**
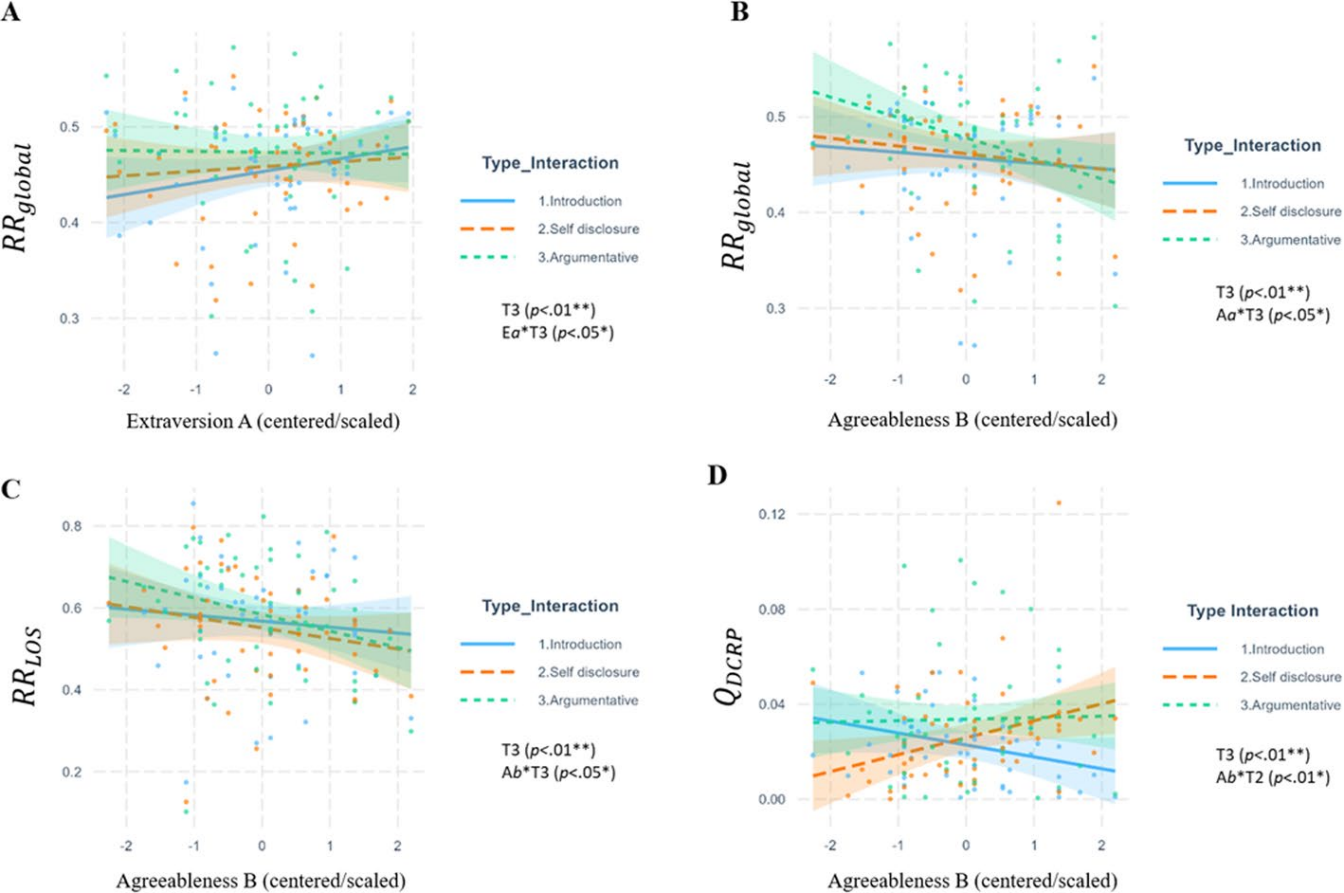
Effects of extraversion on RR_global_ and effects of agreeableness on RR_global,_ RR_LOS_, and Q_DCRP_. *Note*: The plots represent the significant effects of the models of Extraversion and Agreeableness on the variables of speech coordination (*RR*_*global*_*, RR*_*LOS*_*)* and nonverbal interactional dominance (*Q*_*DCRP*_). The significant effects are **A**: [T3. Argumentative (β = 0.32, *t*_(100)_ = 2.98, *p* < .01)], and [Extraversion_A_*T3.Argumentative (β = −0.22, *t*_(100)_ = −2.04, *p* < .05)]; **B**: [T3.Argumentative (β = 0.34, *t*_(100)_ = 3.23, *p* < .01)], and [Agreeableness_B_*T3. Argumentative (β = −0.26, *t*_(100)_ = *−*2.37, *p* < .05)]; in **C**, [Agreeableness_B_*T3.Argumentative (β = −0.18, *t*_(100)_ = *−*2.01, *p* < .05)]; in **B**, [T3.Argumentative (β = 0.50, *t*_(100)_ = 2.81, *p* < .01)], and [Agreeableness_B_*T2.Self-disclosure (β = 0.56, *t*_(100)_ = 3.06, *p* < .01)]

To estimate how personality differences predicted speech dynamics, we fit mixed effects models that revealed how higher scores on Extraversion predicted global speech coordination (*RR*_*global*_) across conversational topics. Having at least one extravert in a dyad predicted lower global speech coordination during argumentative conversations compared to introductions (β = −0.22, *t*(100) = −2.04, *p* < .05; see Table 5, Model 1). Extraverts often had higher speech coordination levels (*RR*_*global*_, see Table 5), indicating attunement in conversations and turn-taking dynamics across all lags, and all conversational topics (aligned to H2a). Conversely, introverted participants exhibited the most significant variation between conversation types, showing the lowest *RR*_*global*_ during introductions and the highest during arguments (see Fig. 6A). Dyads with extraverted participants coordinated smoothly in all conversation topics (high *RR*_*global*_). In our analyses, Extraversion and Agreeableness were considered separately to isolate their unique contributions to speech coordination. This decision was based on both theoretical distinctions between these traits and the empirical observation that their correlation in our sample was weak (*r* = 0.14, *p* = .16). Analyzing them in separate models allowed us to better capture their specific influences without potential over-adjustment or dilution of effects.

**Table 5:** Mixed-effects models predicting CRQA, DCRP, and aCRQA measures from Extraversion and task

Predictors	M1. *RR*_*global*_			M2. *RR*_*LOS*_			M3. *Q*_*DCRP*_			M4. *LAM*_*ARD*_			M5. *TT*_*ARD*_		
	B	β	*t*	B	*β*	*t*	B	*β*	*t*	B	*β*	*t*	B	*β*	*t*
Intercept	0.45	− 0.15	54.34***	0.56	− 0.01	28.8***	0.02	− 0.22	7.70***	0.02	− 0.07	8.55***	0.11	− 0.26	6.65***
Extraversion “*A*” (E_A_)	0.01	0.21	1.45	0.03	0.21	1.44	− 0.00	− 0.01	− 0.06	0.00	0.01	0.05	0.00	0.02	0.14
Extraversion “*B*” (E_B_)	− 0.01	− 0.21	− 1.44	− 0.03	− 0.18	− 1.22	0.00	0.03	0.23	0.00	0.19	1.35	0.01	0.08	0.57
Task 2. Self-disclosure	0.00	0.07	0.69	− 0.01	− 0.10	− 1.09	0.00	0.12	0.70	0.00	0.18	1.10	0.05	0.44	2.51*
Task 3. Argumentative	0.02	0.32	2.98**	0.01	0.09	1.04	0.01	0.54	3.02**	0.00	0.09	0.57	0.05	0.37	2.10*
E_A_ * E_B_	0.01	0.18	1.2	0.02	0.12	0.76	− 0.00	− 0.13	− 0.86	0.00	0.06	0.44	− 0.00	− 0.03	− 0.19
E_A_ * Task 2	− 0.01	− 0.13	− 1.15	− 0.01	− 0.06	− 0.62	0.00	0.16	0.88	0.00	0.00	0.01	0.02	0.14	0.78
E_A_ * Task 3	− 0.01	− 0.22	− 2.04*	0.00	0.00	0.05	− 0.00	− 0.14	− 0.76	− 0.00	− 0.14	− 0.85	0.00	0.00	0.01
E_B_ * Task 2	0.01	0.20	1.77	0.02	0.12	1.28	− 0.01	− 0.34	− 1.82	− 0.00	− 0.16	− 0.93	− 0.00	− 0.03	− 0.18
E_B_ * Task 3	0.00	0.02	0.17	0.02	0.12	1.33	− 0.00	− 0.09	− 0.46	0.00	0.14	0.81	0.00	0.04	0.21
E_A_*E_B_*Task 2	0.00	− 0.04	− 0.36	− 0.01	− 0.09	− 0.91	0.01	0.36	1.82	− 0.01	− 0.42	− 2.32*	− 0.01	− 0.11	− 0.55
E_A_*E_B_*Task 3	0.00	− 0.04	− 0.30	0.00	0.03	0.28	− 0.00	− 0.04	− 0.20	− 0.00	− 0.30	− 1.66	0.00	0.04	0.20
ICC	0.80			0.80			0.13			0.28			0.18		
Marg. R^2^/Cond. R^2^	0.063/0.725			0.050/0.805			0.106/0.218			0.094/0.346			0.063/0.235		
AIC	− 379.1			− 175.4			− 597			− 713.2			− 118.3		

N_i_ = 50 dyads (100 participants); N_t_ = 150 observations (50_i_*3 topics)

B = unstandardized raw estimate; β = beta weights (standardized). Significance is indicated in bold: *p < .05. **p < .01. ***p < .001*. p*-values were BH corrected (Benjamini & Hochberg, 1995) FDR procedure

Task 1 (Introduction) is the baseline. Task 2 = Self-disclosure; Task 3 = Argumentative

E = Extraversion. ICC = Intra-class Correlation Coefficient. AIC = Akaike’s Information Criterion (lower values indicate better fit). Personality traits were centered/scaled. *RR*_*global*_ = Recurrence Rate Global, *RR*_*LOS*_ = Percentage of Speech across the line of Synchrony (lag-zero), *Q*_*DCRP*_ = Quotient Diagonal Cross-Recurrence Profile (absolute), *LAMa* = anisotropic Laminarity, *TTa* = *anisotropic Trapping Time, LAM*_*ARDf*_ = Relative difference of anisotropic Laminarity, *TT*_*ARD*_ = Relative difference of anisotropic Trapping Time. Definitions of each measure can be found in Table 2

In terms of Agreeableness, at least one partner with high scores predicted reduced global speech coordination during argumentative conversations compared to introductions (*RR*_*global*_, see Table 6, Model 1; β = −0.26, *t*(100) = −2.37, *p* < .05). This finding counters H2c and may reflect more silences or instances of simultaneous talking (overlap). Conversely, low Agreeableness predicted the highest dyadic global speech coordination during argumentative conversations (see Fig. 6B), supporting H2e. For the line of synchrony (*RR*_*LOS*_), only Agreeableness predicted significant differences during argumentative tasks versus introductions (β = −0.18, *t*(100) = −2.01, *p* < .05). Here, lower Agreeableness scores predicted strong coordination (*RR*_*LOS*_, see Table 6, Model 2), in support of H2e, which suggests increased turn-taking dynamics, attuned and swift conversational exchanges without delay (at lag-zero).

**Table 6:** Mixed-effects models predicting CRQA, DCRP and aCRQA measures from Agreeableness and task

Predictors	M1. *RR*_*global*_			M2. *RR*_*LOS*_			M3. *Q*_*DCRP*_			M4. *LAM*_*ARD*_			M5. *TT*_*ARD*_		
	B	β	*t*	B	*β*	*T*	B	*β*	*t*	B	*β*	*t*	B	*β*	*t*
Intercept	0.46	− 0.10	55.14^***^	0.57	0.03	29.27***	0.02	− 0.21	7.83***	0.02	− 0.02	8.93***	0.11	− 0.25	6.90***
Agreeableness “*A*” (A_A_)	− 0.00	− 0.02	− 0.17	− 0.01	− 0.09	− 0.62	− 0.00	− 0.04	− 0.30	− 0.00	− 0.12	− 0.81	0.00	0.03	0.18
Agreeableness “*B*” (A_B_)	− 0.01	− 0.09	− 0.67	− 0.01	− 0.10	− 0.73	− 0.01	− 0.23	− 1.67	0.00	0.07	0.48	− 0.00	− 0.12	− 0.82
Task 2. Self-disclosure	0.00	0.07	0.66	− 0.02	− 0.12	− 1.31	0.00	0.14	0.79	0.00	0.10	0.63	0.05	0.42	2.38*
Task 3. Argumentative	0.02	0.34	3.23^**^	0.02	0.12	1.31	0.01	0.50	2.81**	− 0.00	− 0.04	− 0.23	0.05	0.39	2.20*
A_A_ * A_B_	− 0.01	− 0.09	− 0.81	− 0.02	− 0.12	− 1.01	− 0.00	− 0.12	− 1.12	− 0.00	− 0.15	− 1.32	− 0.01	− 0.10	− 0.85
A_A_ * Task 2	− 0.00	− 0.01	− 0.11	0.00	0.02	0.20	− 0.00	− 0.21	− 1.10	0.00	0.32	1.85	0.02	0.17	0.90
A_A_ * Task 3	− 0.01	− 0.09	− 0.77	− 0.00	− 0.02	− 0.17	− 0.00	− 0.15	− 0.80	0.00	0.42	2.44*	0.01	0.12	0.65
A_B_ * Task 2	− 0.00	− 0.04	− 0.36	− 0.01	− 0.08	− 0.87	0.01	0.56	3.06**	0.01	− 0.07	− 0.45	0.01	0.12	0.65
A_B_ * Task 3	− 0.02	− 0.26	− 2.37*	− 0.03	− 0.18	− 2.01^*^	0.01	0.26	1.42	− 0.00	0.29	1.76	− 0.03	− 0.21	− 1.15
A_A_*A_B_*Task 2	− 0.00	− 0.01	0.87	0.00	0.03	0.35	0.00	0.16	1.08	0.00	0.07	0.55	0.00	0.04	0.26
A_A_*A_B_*Task 3	− 0.01	− 0.14	− 1.57	− 0.01	− 0.08	− 1.12	0.00	0.16	1.06	0.00	0.36	2.74**	− 0.01	− 0.04	− 0.29
ICC	0.70	0.79	0.13	0.29	0.16										
Marg. R^2^/Cond. R^2^	0.090/0.727	0.071/0.808	0.130/0.241	0.117/0.377	0.105/0.252										
AIC	− 379.7	− 176.4	− 599.3	− 716.3	− 122.2										

*N*_i_ = *50 dyads (100 participants); N*_*t*_ = *150 observations (50*_*i*_ * 3 topics)

B = unstandardized raw estimate; β = beta weights (standardized). Significance is indicated in bold: *p < .05. ^**^p < .01. ***p < .001. *p* values were BH corrected (Benjamini & Hochberg, 1995) FDR procedure. Task 1 (Introduction) is the baseline. Task 2 = Self-disclosure; Task 3 = Argumentative. A = Agreeableness. ICC = Intraclass Correlation Coefficient. AIC = Akaike’s Information Criterion (lower values indicate better fit). Personality traits were centered/scaled. *RR*_*global*_ = Recurrence Rate Global, *RR*_*LOS*_ = Percentage of Speech across the line of Synchrony (lag-zero), *Q*_*DCRP*_ = Quotient Diagonal Cross-Recurrence Profile (absolute), *LAMa* = anisotropic Laminarity, *TTa* = *anisotropic Trapping Time, LAM*_*ARD*_ = Relative difference of anisotropic Laminarity, *TT*_*ARD*_ = Relative difference of anisotropic Trapping Time. Definitions of each measure can be found in Table 2

In summary, Extraversion significantly predicted global speech coordination across conversational topics, with extraverted individuals showing greater engagement in turn-taking. Introverts exhibited the most pronounced differences, with coordination lowest during introductions and highest during arguments. Agreeableness also impacted speech dynamics, particularly in argumentative contexts, where higher scores correlated with reduced coordination and potentially more overlaps or silences. Overall, while high Extraversion was consistently linked to greater speech coordination, high Agreeableness demonstrated nuanced effects that varied by conversation topic.

### Leader–Follower Dynamics and Nonverbal Interactional Dominance

When exploring the balance of leader–follower dynamics the models based on the Quotient of Diagonal Cross Recurrence Profiles (*Q*_*DCRP*_) exhibited a significant additive effect during the argumentative task in the models of Extraversion (β = 0.54, *t*(100) = 3.02, *p* < .01*,* see Table 5, Model 3) and Agreeableness (β = 0.50, *t*_(100)_ = 2.81, *p* < .01*,* see Table 6, Model 3). During the argumentative conversation, the leader–follower imbalances strengthened (more *Q*_*DCRP*_), thus one of the interacting partners typically took the initiative, e.g., spoke first while the dyadic partner followed those rhythms. There were no significant effects of Extraversion linked to leader–follower dynamics (H3a not supported).

Higher Agreeableness scores predicted more balanced interactions (lower *Q*_*DCRP*_) during the self-disclosure compared to introductions (β = 0.56, *t*_(100)_ = 3.06, *p* < .01). However, during self-disclosure tasks, higher Agreeableness was associated with greater leader–follower imbalances (high *Q*_*DCRP*_) when self-disclosing, and more balanced conversations during the introduction (see Fig. 6D), suggesting that while higher scores promote balance in introductions, they can also facilitate initiating behaviors in self-disclosure contexts (supporting H3b). This indicates that one partner may allow the other to speak or remain silent, effectively leading the interaction.

To visualize the recurrence rates across different lags and leader–follower dynamics, Diagonal Cross-Recurrence Profiles (*DCRPs*) were plotted across the conversation topics (Fig. 5A). These plots showed (descriptively) the highest recurrence percentage at lag-zero (line of synchrony) during the argumentative conversation and the lowest recurrence rates during self-disclosure, indicating behavioral similarity and a strong and immediate response between the participants during arguments. During the introduction, the immediate effect–as captured by lag-zero–does not prominently show the asymmetries, and as the lags increase, asymmetries become more apparent, suggesting that interaction dynamics evolve over time.

When examining DCRPs in relation to personality traits (Fig. 5C), we segmented dyads into low and high personality trait scores using a threshold of ± 0.5 standard deviations. The highest *RR*_*LOS*_ (on the LOS or at lag-zero) was observed in the dyads composed of low/high Agreeableness, indicating a high degree of immediate speech coordination. As time lags increased, there was a trend toward leading-follower dynamics for these dyads, with one individual taking the lead. This pattern was also noted in low and discordant agreeable dyads but was less pronounced. The lowest RR_*LOS*_ was observed in introverted dyads, reflecting lower immediate speech coordination. This segmentation and visualization have only descriptive purposes.

**Fig. 7:**
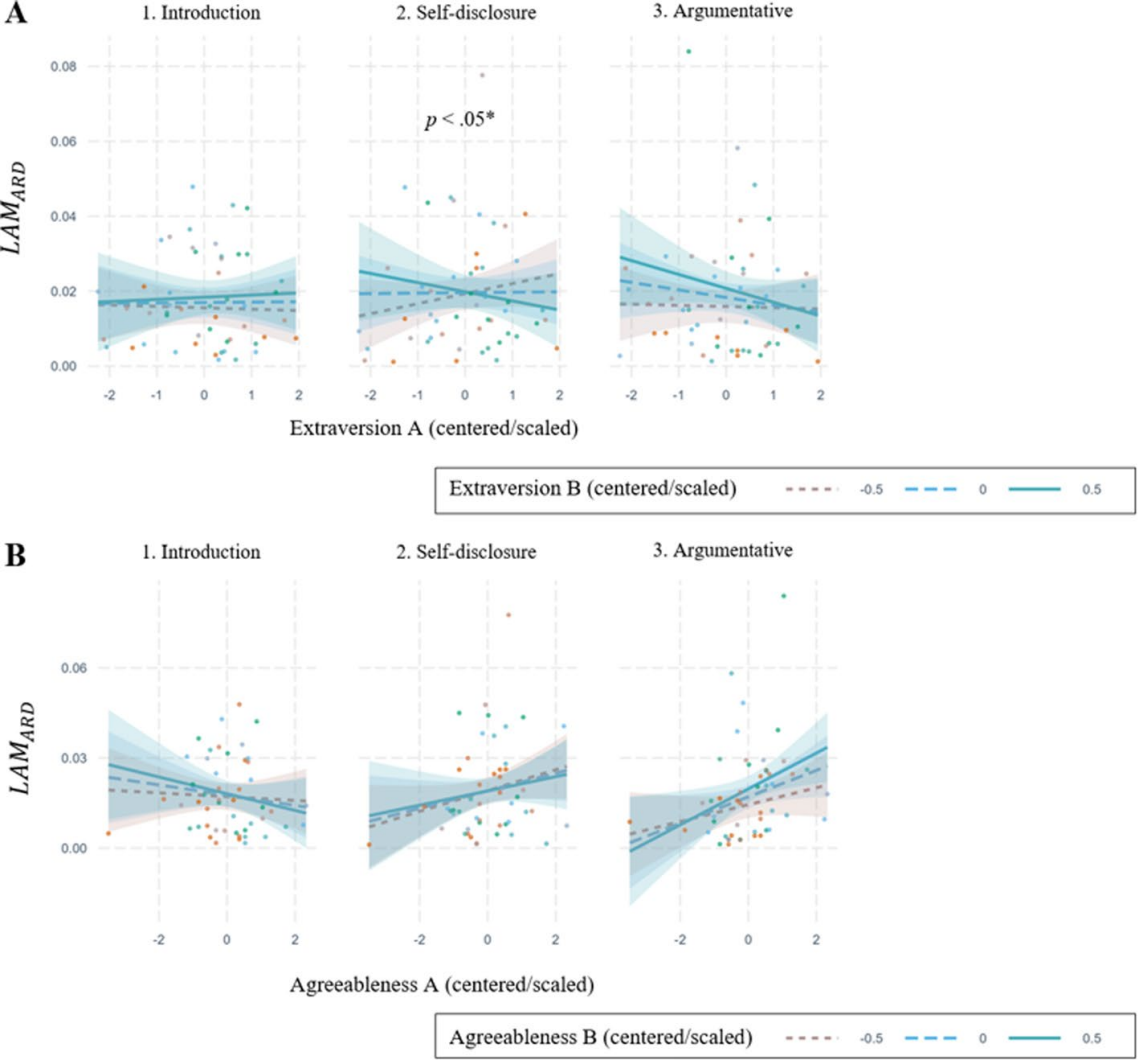
Effects of Extraversion and Agreeableness on LAM_ARD_. The plots show the effects of Extraversion (**A**) and Agreeableness (**B**) on *LAM*_*ARD*_*.* **A** and **B** correspond to separate models (see Tables 6 and 7). **A** the effect of Extraversion_A_*Extraversion_B_ * (2)Self-Disclosure is statistically significant (*β* = −0.42, *t*_(100)_ = −2.32, *p* < .05). **B** the significant effects are Agreeableness_A_* (3)Argumentative (*β* = 0.42, *t*_(100)_ = 2.44, *p* < .05), and Agreeableness_A_* Agreeableness_B_*(3)Argumentative (*β* = 0.36, *t*_(100)_ = 2.74, *p* < .01)

When modeling the effects of personality traits on the relative difference of Laminarity (*LAM*_*ARD*_) the Extraversion scores of both conversational partners significantly predicted *LAM*_*ARD*_ during the self-disclosure task (model 4, Table 5; β = −0.42, *t*_(100)_ = −2.32, *p* < .05), with similarities in Extraversion associated with lower *LAM*_*ARD*_ (Fig. 7A)*.* This suggests that greater personality similarity in Extraversion results in symmetrical conversational dynamics and reduced nonverbal interactional dominance. In contrast, higher asymmetries were found in dissimilar dyads, where one partner tended to dominate nonverbally (aligning with H3a).

Regarding Agreeableness, differences between conversational partners predicted *LAM*_*ARD*_ during the argumentative task (β = 0.36, *t*_(100)_ = 2.74, *p* < .01; see Fig. 7B). Specifically, greater differences in Agreeableness were related to lower asymmetries in nonverbal interactional dominance, while similarity, particularly among highly agreeable individuals, predicted higher asymmetries, indicating stronger influence from one partner's behavior on the other (contradicting H3b).

Finally, regarding Trapping Time *TT*_*ARD*_, no significant effects were linked to personality traits. Only the type of conversation, self-disclosure, and argumentative significantly explained differences in *TT*_*ARD*_ in both models of Extraversion (Table 5, Model 6) and Agreeableness (Table 6, Model 6). In both cases, the average duration of the asymmetries was expected to be higher and last longer during the self-disclosure and argumentative conversations than the introduction.

### Perception of the Interactions (Appraisals)

Finally, we modeled the effects of personality differences (Extraversion and Agreeableness, analyzed separately) of both conversational partners on our speech coordination variables (*RR*_*LOS*_*, Q*_*DCRP*_*,* and *LAM*_*ARD*_) in separate models to assess each of the appraisals reported by participants after their conversations (see the methods section for more details about each model). We corrected *p*-values for multiple hypothesis testing using the Benjamini–Hochberg procedure (1995). The supplementary materials contain detailed tables of all models and figures with significant effects.

#### Inclination for Communication (Need to Communicate with a Partner)

Extraversion scores predicted a higher need to communicate with the interacting partner (β = 0.10, *p* < .05) without interaction from other variables. Both higher Extraversion levels of the interacting partners and increased speech coordination (*RR*_*LOS*_) were associated with an increased perceived need to communicate (β = 0.11, *p* < .01), as indicated by enhanced coordination of speech across the line of synchrony (model 1, Table S1, Figure S1.A). In this context, speech coordination (*RR*_*LOS*_) reflects greater reciprocity and attunement in speech-silence matching, characterized by more time spent talking, fewer pauses, silences, and interruptions. Introverted dyads exhibited the lowest inclination for communication overall. In discordant extroverted dyads (low/high Extraversion), a decrease in speech coordination (*RR*_*LOS*_) was associated with an increased need for communication. For Agreeableness, the interaction effect between the scores of both conversational partners predicted decreases in the need for communication among low agreeable individuals in the *LAM*_*ARD*_ model (β = −0.08, *p* < .05) (model 1, Table S5, Figure S1.B); conversely, the presence of at least one agreeable individual in the dyad (discordant dyads) predicted an increased inclination for communication. No significant effects were found for speech variables and Agreeableness.

#### Using the Partner’s Behavior as a Guide for Own Behavior

Asymmetries in nonverbal interactional dominance were observed in the *LAM*_*ARD*_ model, where lower Extraversion scores were associated with greater perceived behavioral adjustments to partner cues (β = −0.14, *p* < .01) (model 2, Table S2, Figure S2.A). An interaction effect between Extraversion scores (of one interacting partner) and *LAM*_*ARD*_ indicated that lower Extraversion scores were associated with higher asymmetries (higher *LAM*_*ARD*_) and predicted increased perceived behavioral use of partner cues (β = 0.27, *p* < .05). A three-way interaction also showed that Extraversion scores of both partners (β = 0.23, *p* < .05) predicted lower symmetry (lower *LAM*_*ARD*_) in discordant dyads. The perceived alignment to partner cues was more pronounced among introverts, whereas high asymmetry (high *LAM*_*ARD*_) led participants to align their behavior with their partners’ behavior, particularly in discordant (low/high) and extroverted dyads, indicating a more pronounced nonverbal interactional dominance.

In the Agreeableness model with *LAM*_*ARD*_ as a predictor, the interactive three-way effect (β = 0.27, *p* < .05) suggested that during lower nonverbal interactional dominance (lower *LAM*_*ARD*_), highly agreeable dyads were more likely to use partner behavior as a guide for their own. However, in discordant agreeable dyads, the interactive effects indicated that agreeable individuals exhibited initiating behaviors, while disagreeable individuals aligned their behavior with partner cues when *LAM*_*ARD*_ was higher. This suggests that highly agreeable dyads tend to utilize partner behaviors, contributing to symmetrical interactions (lower *LAM*_*ARD*_). In contrast, agreeable individuals may take the initiative when interacting with disagreeable partners, while disagreeable individuals align their behaviors with partner cues during higher *LAM*_*ARD*_ (nonverbal interactional dominance asymmetries).

#### Attempts to Lead the Conversation

The Agreeableness of at least one conversational partner and the (im)balances in the conversation (*Q*_*DCRP*_) significantly predicted perceived attempts to lead the conversation. Increases in imbalances in leading-following dynamics (*Q*_*DCRP*_) predicted an increased perceived attempt to lead the conversation by low agreeable individuals (β = 0.17, *p* < .05). A three-way interaction involving the Agreeableness scores of both conversational partners and the interaction balance (*Q*_*DCRP*_) indicated that in dissimilar dyads (low/high), higher imbalances (*Q*_*DCRP*_) predicted increases in perceived attempts to lead by low agreeable individuals (β = −0.32, *p* < .05) (model 3, Table S6, Figure S3).

#### “Smooth, Natural, and Relaxed” Conversations

Agreeableness positively predicted reports of smooth, natural, and relaxed conversations when considered without interaction from other variables (*p* < .05) (model 6, Table S4, Figure S4.A). However, the interaction between Agreeableness and speech coordination (*RR*_*LOS*_) was negatively related to perceptions of the conversation as smooth, natural, and relaxed (β = −0.35, *p* < .05). This suggests a trade-off effect, where while Agreeableness and speech coordination (at lag-zero) may enhance aspects of a conversation (e.g., interpersonal attunement), they could also contribute to a perception of less relaxation and naturalness, which will be discussed further in the discussion section.

#### Felt Accepted and Respected by Partner

Overall, a main effect indicated that higher Extraversion scores were associated with increases in perceptions of being accepted and respected by the interacting partner (β = 0.25, *p* < .01); lower scores on Extraversion predicted decreases in this perception (model 9, Table S2). No significant effects were found for Agreeableness and speech coordination variables, implying that these factors might not directly influence feelings of acceptance and respect in conversation, or their effects could be more subtle or complex.

#### Desire to Interact More with Partner in the Future

Only Extraversion and speech coordination (*RR*_*LOS*_)—such as speech-silence attunement, reciprocity, and fewer silences and interruptions—increased participants’ willingness for future interactions (model 10, Table S1, Figure S4.B). High Extraversion scores of at least one interacting partner and increased speech coordination predicted a higher post-conversational desire to interact in the future (β = 0.46, *p* < .01). This implies that both personality traits and conversation dynamics significantly influence the desire for future interactions.

#### Enjoyment of the Interaction

High Extraversion (of at least one interacting partner) and higher speech coordination (*RR*_*LOS*_) were associated with increased enjoyment of the interactions (β = 0.28, *p* < .05) (model 11, Table S1). Conversely, for introverted individuals, higher speech coordination predicted decreased enjoyment. The effect of both conversational partners was significant (three-way interaction), with highly extroverted dyads enjoying conversations with higher speech coordination more (β = 0.42, *p* < .05) (Figure S5.A). In more dissimilar dyads (extroverted/introverted), lower speech coordination was linked to increased enjoyment. The Extraversion of both interacting partners and asymmetries in nonverbal interactional dominance (*LAM*_*ARD*_) showed that lower asymmetries and high Extraversion scores of both interacting partners predicted increased enjoyment (β = −0.25, *p* < .05) (model 11, Table S2). When asymmetries (*LAM*_*ARD*_) increased, enjoyment increased for discordant dyads (introverted/extroverted). Furthermore, introverted dyads were associated with decreased enjoyment compared to other participants, regardless of other interpersonal speech dynamics in the conversation. Regarding balance in leader–follower dynamics (*Q*_*DCRP*_), a three-way interaction indicated that increased imbalances —where one partner tended to initiate/lead or act first, either by speaking or being silent— were predictive of enjoyment in extroverted dyads (β = 0.44, *p* < .05), while in discordant dyads (introverted/extroverted), more balanced interactions (*Q*_*DCRP*_) were predictive of increased enjoyment (model 11, Table S3, Figure S5.C).

#### Perceived Partner as Likable

The main effect of Extraversion indicated that increases in this trait were positively associated with increases in the report of liking the conversational partner (β = 0.14, *p* < .01) (model 14, Table S1, Figure S6.A). Similarly, the interaction effect between Extraversion scores and increases in speech coordination (*RR*_*LOS*_) predicted increased reports of liking the conversational partner (β = 0.49, *p* < .05). Conversely, decreased scores on Extraversion and lower values of speech coordination predicted increases in liking the other person. The three-way effect between the Extraversion scores of both partners and speech coordination suggested that in extroverted dyads, increases in speech coordination were associated with liking the interacting partner to a greater extent, whereas in discordant dyads (introverted/extroverted), decreases in speech coordination (indicating silences) were associated with liking the other person (β = 0.21, *p* < .05) (model 14, Table S1). These effects may indicate that introverted and extroverted individuals valued different aspects of the conversation. Extraverts seemed to appreciate a more dynamic conversation, while introverts may find value in moments of silence or pauses during interactions. The distinction in preferences between introverted and extroverted individuals is particularly salient in discordant dyads.

#### Perceived Partner as Empathic and Understanding

Finally, increased Extraversion scores were associated with a heightened perception of the conversational partner as empathic and understanding (main effect, β = 0.02, *p* < .05). Additionally, the interaction between Extraversion and nonverbal interactional dominance (*LAM*_*ARD*_) revealed that lower asymmetries and higher Extraversion predicted greater perceived empathy and understanding, while lower Extraversion scores coupled with increased asymmetries were linked to higher perceptions of empathy (β = −0.31, *p* < .05) (model 15, Table S2, Figure S7). These results reflect how personality differences shape appraisals of interpersonal dynamics in conversation. For other appraisal variables, no significant effects related to personality traits were identified after correcting for multiple hypotheses testing; full details can be found in the supplementary materials. Overall, these findings emphasize the connection between personality traits, speech coordination, and perceptions of interaction dynamics, highlighting the importance of considering individual communication styles when evaluating social interactions.

## Discussion

This study aimed to achieve four goals: (1) to explore the effect of high-level constraints (specifically, conversational topics) on interpersonal speech coordination, leading-following dynamics, and nonverbal interactional dominance in dyadic conversations; (2) to examine how these speech coordination structures relate to the socially relevant personality traits of Extraversion and Agreeableness; (3) to investigate how these personality traits influence speech coordination, leader–follower dynamics, and nonverbal interactional dominance; and (4) to assess the impact of speech coordination, dynamics, and personality traits on appraisals of the interactions reported by the conversational partners. Below, we discuss our key findings, considering their theoretical implications, limitations, and future directions.

### Interpersonal Speech Coordination and Conversation Topic

We hypothesized that different conversational topics would explain variations in speech coordination (*RR*_*global*_, *RR*_*LOS*_) (H1a)*,* leader–follower dynamics (*Q*_*DCRP*_*,*) and nonverbal interactional dominance (*LAM*_*ARD*_*,* and *TT*_*ARD*_) (H1b). Our findings supported that conversational topics significantly influenced global speech coordination, with increased coordination observed during argumentative conversations compared to introductory ones. Similarly, leader–follower dynamics and nonverbal interactional dominance were affected, with larger asymmetries noted in argumentative and self-disclosure conversations, aligning with our expectations and existing literature on interpersonal coordination. Studies indicate that the dynamic properties of interpersonal interactions are shaped by situational constraints (Fusaroli et al., 2014), reinforcing the view of language and joint action as complex adaptive systems (Arellano-Véliz et al., 2024b; Ellis & Larsen-Freeman, 2009; Paxton & Dale, 2017; Tschacher et al., 2018).

The role of the high-level constraints (e.g., conversation topics) in speech coordination can vary as it flexibly adjusts to casual encounters, bonding/affiliating, or competitive goals (Paxton & Dale, 2017). Previous research has shown that nonverbal interactional dominance patterns can impact interaction quality as participants become sensitive to distinct conversational cues beneficial for interpersonal goals (Reuzel et al., 2014). In our study, argumentative conversations led to significantly higher global speech coordination and larger asymmetries in leader–follower dynamics, reflecting competitive interactional settings (Arellano-Véliz et al., 2024b; Tschacher et al., 2018). Other studies reported that in-phase (simultaneous) bodily coordination decreased during arguments (Paxton & Dale, 2013). However, speech coordination involves compensatory dynamics (that do not necessarily unfold simultaneously) and supports the emergence of functions or the achievement of goals (Nowak et al., 2017).

Our analysis of speech coordination focused on reciprocity in turn-taking and conversational rhythm rather than simultaneous performance (Reuzel et al., 2013). Reciprocity is crucial in argumentative contexts, fostering a dynamic exchange of arguments. Furthermore, the greater relative difference in trapping time (*TT*_*ARD*_) during self-disclosing and argumentative conversations suggests prolonged influence by one partner, enabling opportunities for reciprocal interaction (Worgan & Moore, 2010). Even though it can indicate more “control” in the dynamics, this can also afford a *reciprocal* interaction when self-disclosing. According to previous research, therapists use their nonverbal interactional skills to intensify the attunement with clients by leading in the use of turn-taking and driving dynamical coordination of speech (Reuzel et al., 2014). This type of behavior might have improved the communicational rhythm when self-disclosing, involving longer speech coordination.

While conversational topics did not correlate with speech coordination across the line of synchrony (*RR*_*LOS*_) or one measure of nonverbal dominance (*LAM*_*ARD*_), these findings warrant replication in larger samples, given the modest size of our study. In this sense, some responses and exchanges might be time-sensitive and be visible at longer lags rather than simultaneously or very close in time, as indicated by speech coordination across the line of synchrony (*RR*_*LOS*_), which represents swift dynamics*.*

### Personality Traits, Speech Coordination, and Nonverbal Interactional Dominance

We argued that Extraversion and Agreeableness would explain some variability in dyadic speech coordination (H2), leader–follower dynamics, and nonverbal interactional dominance (H3), based on prior work on body motion in interpersonal dynamics (Arellano-Véliz et al., 2024b).

Extraversion scores were associated with increased speech coordination (*RR*_*global*_) while introverts coordinated less (H2a). Extraverted individuals showed more coordinated communication across multiple time lags and all conversational topics, suggesting less context dependency. Introverts exhibited the lowest speech coordination, especially during introductions; they varied in coordination across topics, as argumentative conversations exhibited the highest speech coordination. These results align with the social reactivity hypothesis (Lucas & Diener, 2001), suggesting that extraverts get more pleasure from social interactions, being highly social, gregarious, and outgoing individuals (Costa & McCrae, 1995; Larsen et al., 2025); which promotes intersubjective attunement (Stern, 1985/2018; Harris et al., 2017). Generally speaking, introverts prefer solitude and tend to be more comfortable with their inner worlds, thoughts, and feelings than extroverts (Burger, 1995; Tuovinen et al., 2020).

Regarding the composition of our dyads, we anticipated that at least one extroverted partner would enhance speech coordination (H2b). Our findings support this, as the presence of an extrovert led to increased coordination, which might be relevant to facilitating social interactions for introverts, as it might boost their social engagement (Tuovinen et al., 2020). The interactive effect of both conversational partners was not significant in the models of speech coordination (*RR*_*global*_, and *RR*_*LOS*_), only the individual effect of the trait Extraversion in global speech coordination.

We expected high agreeable scores to associate increased speech coordination and inverse expectation for low-agreeable (or “disagreeable”) dyads (H2c). Dyadic composition was expected to affect speech coordination, especially the presence of a disagreeable individual in the dyad, but it was not supported (H2d). However, the task sensitivity effect we predicted regarding the argumentative conversation was supported (H2e) as low Agreeableness associated with higher speech coordination during the argumentative conversation (*RR*_*global*_ and *RR*_*LOS*_). We argued that this effect could be functional to goal achieving in competitive settings by low-agreeable individuals (e.g., DeYoung, 2015). However, these dynamics might not be positive for the intersubjective attunement of the interacting partners, since the literature suggests that low-agreeable individuals exhibit poor social relationships (Anderson et al., 2020), low concern for others' needs and desires, and less efficient social information processing (e.g., DeYoung et al., 2010).

Concerning *leader–follower dynamics and nonverbal interactional dominance*, we expected that higher scores of Extraversion would be associated with asymmetries in the speech dynamics when interacting with introverts due to leading (initiating) and influencing tendencies (H3a). This was reflected by the relative difference of Laminarity (*LAM*_*ARD*_) when self-disclosing, where personality similarity fostered symmetric speech dynamics. It is possible that extroverted individuals took a leading and initiating role within the conversation allowing for longer periods of interactional attunement.

We anticipated more balanced leading-following dynamics for agreeable individuals compared to disagreeable ones, with the latter potentially leading to larger imbalances (H3b). This was supported by diagonal cross-recurrence profiles (*Q*_*DCRP*_), showing that low-agreeable individuals exhibited larger imbalances during introductions but less so when self-disclosing. In contrast, highly agreeable individuals demonstrated lower imbalance during introductions and higher during self-disclosures, indicating they may take the lead in such conversations.

As for nonverbal interactional dominance (*LAM*_*ARD*_), argumentative conversations elicited greater dominance, particularly among high-agreeable individuals and in discordant dyads (agreeable/disagreeable). Agreeableness, characterized by cooperation and altruism (DeYoung, 2015; Hovhannisyan & Vervaeke, 2022), suggests that highly agreeable partners may take initiative during self-disclosures, fostering a reciprocal and cooperative dynamic (Worgan & Moore, 2010). This tendency is especially pronounced in argumentative contexts, where highly agreeable individuals may dominate the conversation, enhancing attunement. Such dominance should not carry negative connotations; it likely reflects a higher metacognitive capacity in agreeable individuals, enabling them to better understand others’ needs and intentions (DeYoung et al., 2010).

### Perception of the Interaction (Appraisals), Interpersonal Speech Dynamics, and Personality Traits

We predicted that higher dyadic speech coordination (H4a) and symmetrical interactions (H4b) would enhance positive post-conversational appraisals, with extroverted (H4c) and agreeable individuals (H4d) likely rating interactions more positively following coordinated exchanges. The results revealed differentiated effects based on dyadic composition and speech dynamics.

We observed a logical connection between speech dynamics and participants’ appraisals. In the case of Extraversion, this trait consistently predicted positive perceptions of the interaction as expected. Extroversion consistently predicted positive perceptions of interactions, as higher Extraversion scores and increased speech coordination (*RR*_*LOS*_), were associated with positive appraisals of smooth, natural, and relaxed conversations, desire to interact in the future, enjoyment, liking the conversational partner, and inclination to communicate. This indicates that attuned and rapid conversations are particularly valuable for extroverts. *RR*_*LOS*_ reflects swift turn-taking with minimal silence or interruptions. In contrast, introverts may prefer pauses and silences over continuous attunement, aligning with H4a and H4c.

Increased nonverbal interactional dominance (i.e. increases in *LAM*_*ARD*_) led introverts to use their partner's behavior as a guide, reflecting an alignment with partner cues. For discordant extroverted dyads, higher asymmetries in nonverbal dominance enhanced enjoyment, suggesting that such dominance might improve interaction quality presumably by affording and facilitating opportunities for sustaining interactional attunement.

These dynamics may reflect higher intersubjective attunement and relatedness (Stern, 1985/2018). Previous studies indicated that Extraversion predicts subjective well-being in students over four years (Harris et al., 2017), with positive social experiences and feelings of belonging being particularly relevant for young populations. Introverts can also benefit from interactions with dissimilar individuals, as they may require guidance and initiating behaviors to sustain social interactions (Tuovinen et al., 2020). Moreover, introverts might prefer slower conversations with more pauses, given their lower need for social stimulation (DeYoung, 2015; Hovhannisyan & Vervaeke, 2022).

In highly agreeable dyads, agreeable individuals used their partner's behavior as a guide, reflected in lower asymmetries in nonverbal dominance. Agreeableness fosters interpersonal attunement (Anderson et al., 2020), indicating high dyadic and nonverbal coupling. Contrary to our expectations (H4d), increased speech coordination and Agreeableness predicted lower perceived naturalness in conversations. This suggests a trade-off in maintaining coordinated communication—characterized by smooth turn-taking and fewer silence episodes—where highly agreeable individuals may achieve a communicational rhythm without feeling it is natural. They may also require more time to respond or value silence. Previous studies indicate that sustaining coordination can reduce self-regulation of affect (Galbusera et al., 2019), suggesting that Agreeableness' drive for altruism and social harmony (DeYoung, 2015) might lead to greater effort in maintaining smooth dynamics, potentially at the cost of enjoyment. Finally, we observed task sensitivity in both Extraversion and Agreeableness, emphasizing the situational context's role in shaping communication.

### Limitations, Strengths, and Future Directions

Our study has several limitations that warrant consideration. The modest sample size may limit the generalizability of our findings, as some significant effects became non-significant after corrections for multiple tests. Additionally, the predominance of female participants restricts gender comparisons. Future research should aim for larger, more diverse samples to enhance generalizability and replication. In this study, Extraversion and Agreeableness were analyzed separately to examine their distinct contributions to speech coordination. While our decision was guided by theoretical and statistical considerations—ensuring that each trait’s unique effects were not diluted or obscured—we acknowledge that this approach may not fully account for their potential interdependence. Although our data indicate a weak correlation between these traits (*r* = 0.14, *p* = 0.16), future research could explore their combined effects more explicitly, either by including both traits in the same model or using methods such as the Actor-Partner Interdependence Model (APIM), which would require a larger sample size to ensure reliable estimates (e.g., Cook & Kenny, 2005; Kenny et al., 2006). A more comprehensive approach to personality interactions, incorporating all dimensions of the Big Five, may provide further insight into how personality traits jointly shape conversational dynamics.

The use of a balance board for face-to-face interactions may also impact ecological validity, as this setup is not reflective of natural interactions. It would be beneficial to compare the ecological validity of different experimental setups (e.g., laboratory vs. natural settings). Furthermore, the confounding of task order and conversation topics—where argumentative conversations followed introductions—might have influenced our findings, with increased coordination possibly stemming from familiarity rather than the task nature.

Despite these limitations, our employment of nonlinear time-series analysis and experimental methods represents a strength, revealing subtle interpersonal dynamics that might not have been detected otherwise. This approach is a valuable tool for studying interpersonal dynamics and personality. Finally, while our study focused on turn-taking behaviors and key social personality traits, it did not account for the content of conversations or other personality traits. Future research should investigate speech coordination at the content level to gain deeper insights into interpersonal dynamics, communication, and personality.

## Conclusion

Overall, our results emphasize the dynamic interplay between the personality traits Extraversion and Agreeableness, situational constraints (conversation topics), interpersonal speech dynamics –coordination, leader–follower dynamics, and nonverbal interactional dominance–, as well as the subjective experiences emerging from social interactions. Personality traits exhibited relevance in speech dynamics and appraisals, and differences in terms of the dyads’ constitutions were observed. Generally, we observed that extroverted individuals engaged in more coordinated communication across various conversational topics, contrary to introverts. Besides, interpersonal speech coordination seemed to foster intersubjective attunement and positive appraisals in extroverts. Increased speech coordination and Agreeableness were predictive of decreased perceived naturality in the conversations, suggesting a potential trade-off effect. In terms of the methods employed, we were able to observe how situational constraints and personality traits were predictive of interpersonal speech dynamics in the conversations and appraisals. The nonlinear time-series techniques employed exhibited a useful and robust tool for studying interpersonal dynamics in conversations. Our results support the use of dynamical approaches to, not only understanding interpersonal communication, but also its relation to psychological constructs.

## Supplementary Information

Below is the link to the electronic supplementary material.

Supplementary file1 (DOCX 14 KB)

Supplementary file2 (DOCX 1665 KB)

Supplementary file3 (DOCX 47 KB)

## Data Availability

Data availability Further materials such as data can be accessed at https://doi.org/10.17605/OSF.IO/53NZ2.

## Footnotes

1 Since this study is part of a larger data collection and a subsample was considered, we delineate our hypotheses based on Arellano-Véliz et al. (2024b) which focused on coordination and coupling of body motion, as well as pioneer literature by Funder and Sneed (1993), Cuperman and Ickes (2009) and Tschacher et al. (2018).

2 Mixed-effects models were preferred to assess the effects of task conditions alone, ensuring a clear understanding of the impact of the task preserving the dyadic structure, improving generalization by accounting for fixed and random effects (see Bates et al., 2015). Subsequently, the personality predictors were modeled in conjunction with the task conditions. This approach helps isolate the effects of the task conditions from the variables included in the subsequent models.

3 The models followed the structure (‘lme4’ R package): [*RR*_*LOS*_ ~ (Extraversion A * Extraversion B) * Task + (1|Dyad)]. See Bates et al. (2015) to consult the syntax structure and package details. In this example, Extraversion A and B correspond to the scores of each interacting partner. The same procedure was employed for the other response variables and Agreeableness. See equations in the supplementary materials.

4 The models followed the structure (example): Perception of Interaction Variable 1 ~ *RR*_*LOS*_ * (Extraversion A * Extraversion B).

5 The BH procedure controls the false discovery rate (FDR) by ranking p-values from smallest to largest and comparing each to a threshold adjusted for its rank (Benjamini & Hochberg, 1995). This method allows for a flexible balance between detecting true effects and limiting false positives compared to more conservative methods like Bonferroni. Particularly in studies with several hypotheses, as it provides a less stringent threshold for declaring significance while still controlling the expected proportion of false positives. It is also less conservative for small samples. Therefore, BH strikes a better balance by controlling FDR, reducing the likelihood of Type II errors while still addressing the issue of false positives (Benjamini & Hochberg, 1995).
