# Supplementary material for: Beyond Words: Speech Coordination Linked to Personality and Appraisals
Source: J Nonverbal Behav. 2025 Mar 8;49(1):85–123. doi: 10.1007/s10919-025-00482-3 (PMC11982161; doi:10.1007/s10919-025-00482-3)
Supplement: Supplementary file 1 — Supplementary file1 (DOCX 14 KB) [file 10919_2025_482_MOESM1_ESM.docx]

**Supplement 3: Equations**

1. Equation of the model speech coordination at lag-zero (*RR_LOS_*) with Extraversion as predictor (employing the R package “lme4”; Bates et al., 2015), given the formula:

$${RR}_{LOS} \sim((Extraversion.A x Extraversion.B)* Topic +(1|Dyad)$$

Equation:

$${RR}_{LOS i} \sim(N\left( \alpha j\left[ i \right]+ \beta1\left( Topic 2.SelfDisclosure \right)+ \beta2\left( Topic 3.Argumentative \right), \sigma2 \right)$$

$\alpha j \sim N (\gamma\alpha0 + \gamma\alpha1(Extraversion.A)+ \gamma\alpha2\left( Extraversion.B \right)+ \gamma\alpha3\left( Extraversion.A x Extraversion.B \right)+ \gamma\alpha4(Extraversion.A x Topic 2.SelfDisclosure) + \gamma\alpha5(Extraversion.A x Topic 3.Argumentative) + \gamma\alpha6(Extraversion.B x Topic 2.SelfDisclosure) + \gamma\alpha7(Extraversion.B x Topic 3.Argumentative) + \gamma\alpha8(Extraversion.A x Extraversion.B x Topic 2.SelfDisclosure) + \gamma\alpha9\left( Extraversion.A x Extraversion.B x Topic 3.Argumentative \right)\sigma2\alpha j), for Dyad j = 1,\ldots,J$)

*Note:* The response variable *RR_LOS_* refers to speech coordination at lag-zero. Fixed effects were calculated for the Extraversion scores of each participant, and random effects were calculated for the variable “dyad”. The dyadic structure was preserved by computing the interaction between the scores on Extraversion of each interacting partner. The conversational topic “introduction” was the baseline category. The models of *RR_av_,* *Q_LOS_*, *LAM_ARD_*, and *TT_ARD_* follow the exact same structure but with these variables as response variables respectively.

2. Equation of the model speech coordination at lag-zero (*RR_LOS_*) with Agreeableness as predictor (employing the R package “lme4”; Bates et al., 2015), given the formula:

$${RR}_{LOS} \sim((Agreeableness.A x Agreeableness.B)* Topic +(1|Dyad)$$

Equation:

$${RR}_{LOS i} \sim(N\left( \alpha j\left[ i \right]+ \beta1\left( Topic 2.Self-disclosure \right)+ \beta2\left( Topic 3.Argumentative \right), \sigma2 \right)$$

$\alpha j \sim N (\gamma\alpha0 + \gamma\alpha1(Agreeableness.A)+ \gamma\alpha2\left( Agreeableness.B \right)+ \gamma\alpha3\left( Agreeableness.A x Agreeableness.B \right)+ \gamma\alpha4(Agreeableness.A x Topic 2.SelfDisclosure) + \gamma\alpha5(Agreeableness.A x Topic 3.Argumentative) + \gamma\alpha6(Agreeableness.B x Topic 2.SelfDisclosure) + \gamma\alpha7(Agreeableness.B x Topic 3.Argumentative) + \gamma\alpha8(Agreeableness.A x Agreeableness.B x Topic 2.SelfDisclosure) + \gamma\alpha9\left( Agreeableness.A x Agreeableness.B x Topic 3.Agreeableness \right)\sigma2\alpha j), for Dyad j = 1,\ldots,J$)

*Note:* The response variable *RR_LOS_* refers to speech coordination at lag-zero. Fixed effects were calculated for the Extraversion scores of each participant, and random effects were calculated for the variable “dyad”. The dyadic structure was preserved by computing the interaction between the scores on Extraversion of each interacting partner. The conversational topic “introduction” was the baseline category. The models of *RR_av_,* *Q_LOS_*, *LAM_ARD_*, and *TT_ARD_* follow the exact same structure but with these variables as response variables respectively.
