# Supplementary material for: Beyond Words: Speech Coordination Linked to Personality and Appraisals
Source: J Nonverbal Behav. 2025 Mar 8;49(1):85–123. doi: 10.1007/s10919-025-00482-3 (PMC11982161; doi:10.1007/s10919-025-00482-3)
Supplement: Supplementary file 2 — Supplementary file2 (DOCX 1665 KB) [file 10919_2025_482_MOESM2_ESM.docx]

**Supplement: Figures**

**Figure S.1.**

*Need to communicate predicted by Extraversion, RR_LOS,_ and Agreeableness*


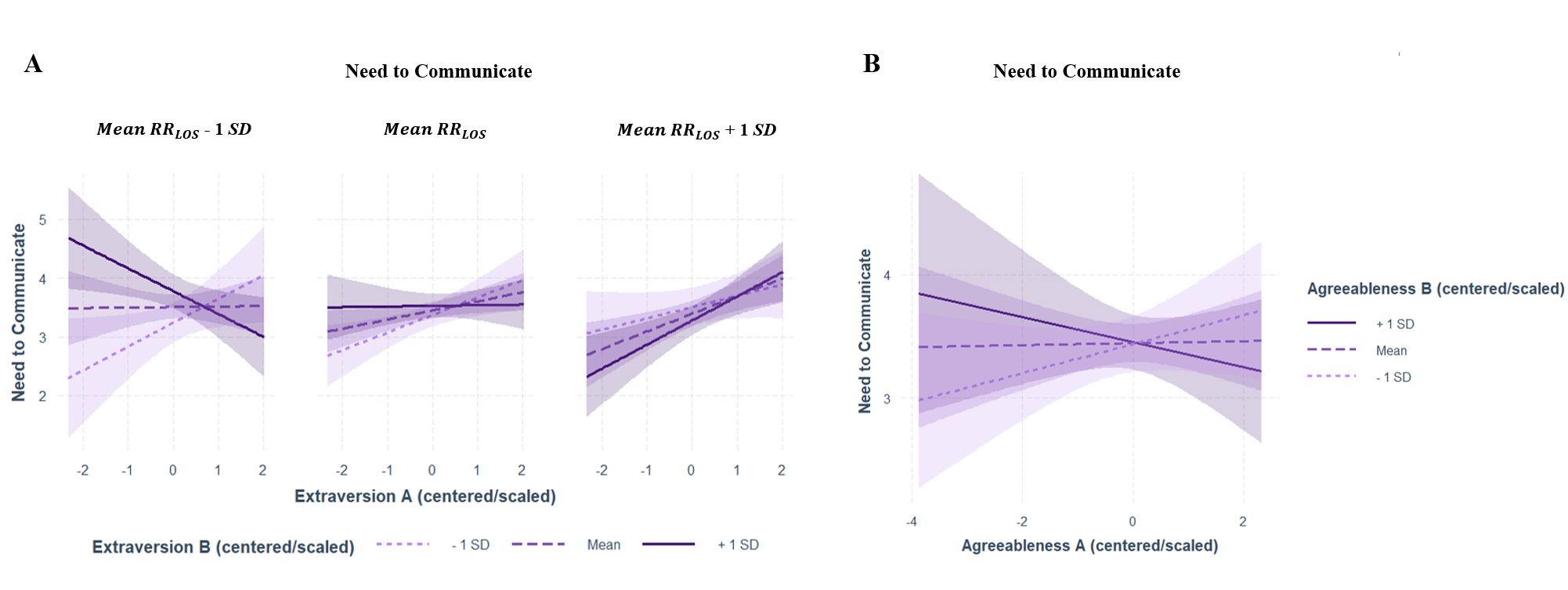


*Note:* Panel A shows the effects of the Extraversion scores of both conversation partners and *RR_LOS_* (-1SD, mean, and +1SD) on the reported need to communicate (*y-axis*) (Table S1, Model 1). Panel B shows the effects of the Agreeableness scores of both conversation partners on the reported need to communicate (*y-axis*) (Table S4, Model 1).

**Figure S.2.**

*Use of the partner’s behavior as a guide predicted by Extraversion, LAM_ARD,_ and Agreeableness*


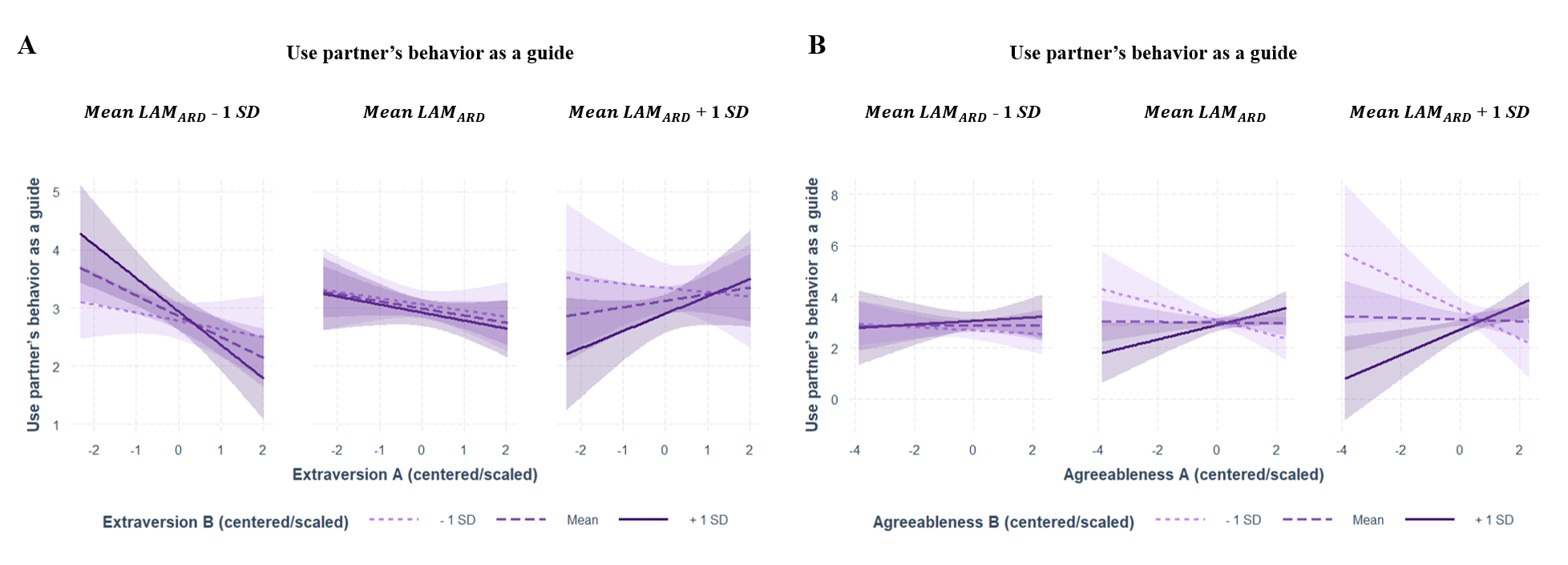


*Note*: Panel A shows the effects of the Extraversion scores of both conversation partners and *LAM_ARD_* (-1SD, mean, and +1SD) on the reported use of the partner’s behavior as a guide (*y-axis*) (Table S2, Model 2). Panel B shows the effects of the Agreeableness scores of both conversation partners and *LAM_ARD_* (-1SD, mean, and +1SD) on the reported use of the partner’s behavior as a guide (*y-axis*) (Table S5, Model 2).

**Figure S.3.**

*Attempt to lead the conversation predicted by Agreeableness and Q_DCRP_*


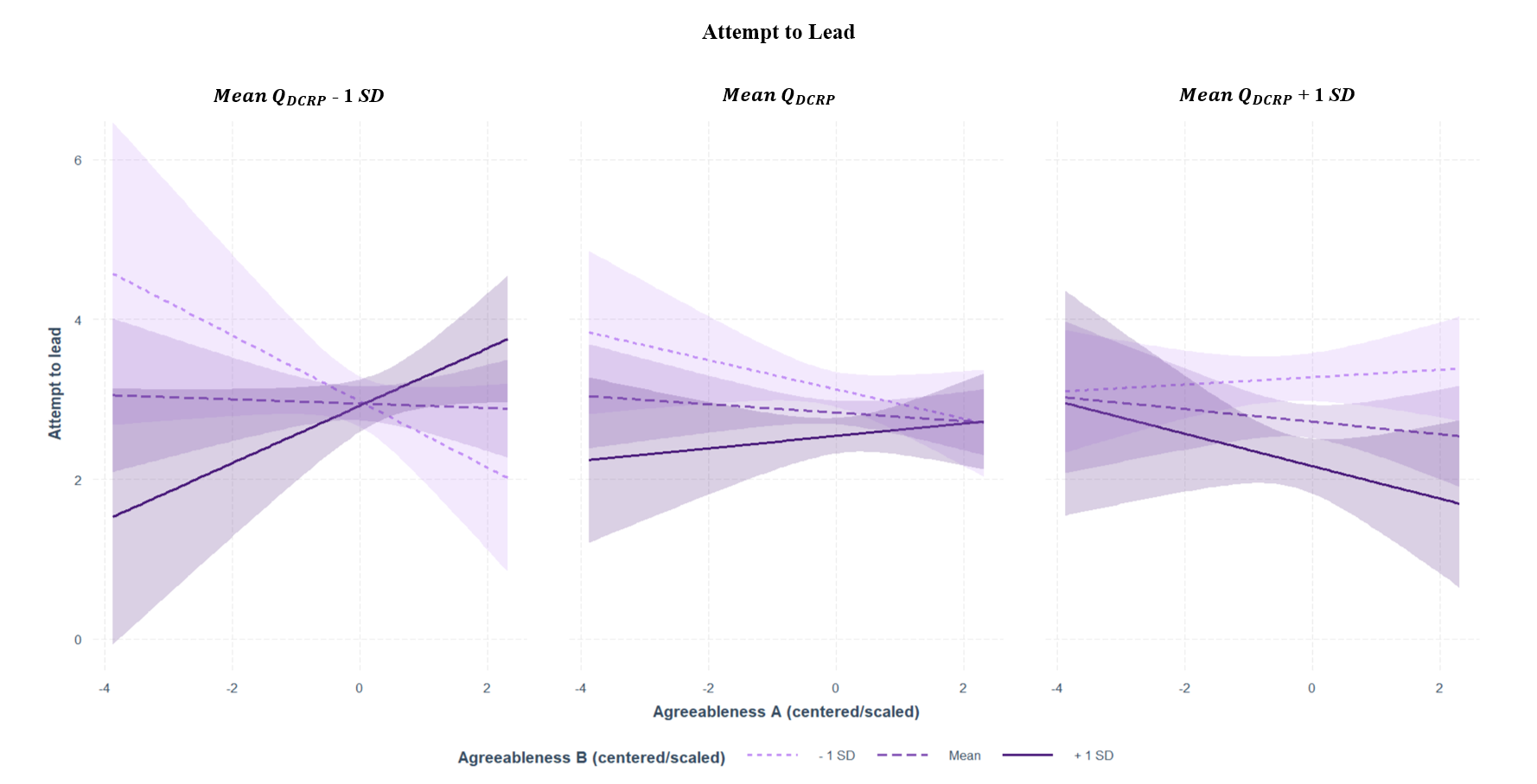


*Note*: The figure shows the effects of the Agreeableness scores of both conversation partners and *Q_DCRP_* (-1SD, mean, and +1SD) on the reported attempt to lead the conversation (*y-axis*) (Table S6, Model 3).

**Figure S.4.**

*Interaction perceived as smooth/natural/relaxed predicted by Agreeableness and RR_LOS,_ and the reported Desire to interact in the future predicted by Extraversion*


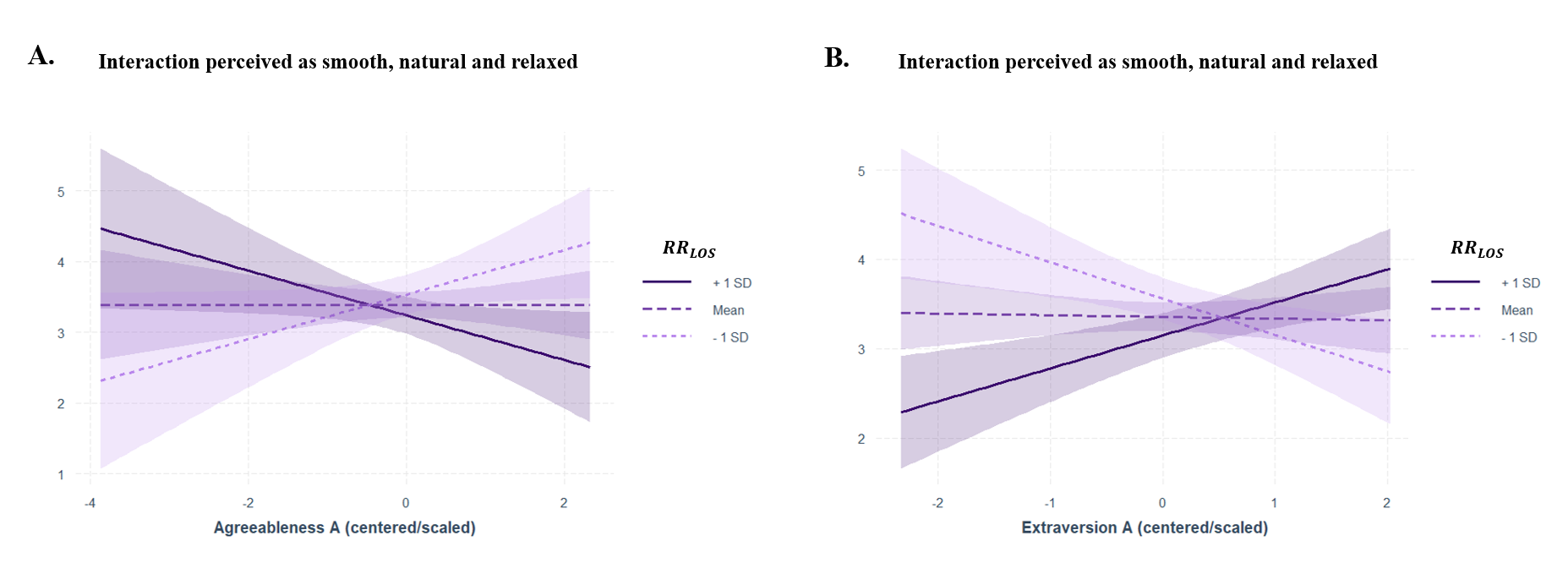


*Note*: Panel A shows the effects of the Agreeableness of one conversation partner and *RR_LOS_* (-1SD, mean, and +1SD) on the reported interaction perceived as smooth/natural/relaxed (*y-axis*) (Table S4, Model 6). Panel B shows the effects of the Extraversion scores of one conversation partner and *RR_LOS_* (-1SD, mean, and +1SD) on the reported desire to interact in the future (*y-axis*) (Table S1, Model 10).

**Figure S.5.**

*Enjoyment of the interaction predicted by Extraversion, RR_LOS_, LAM_ARD_ and Q_DCRP_*


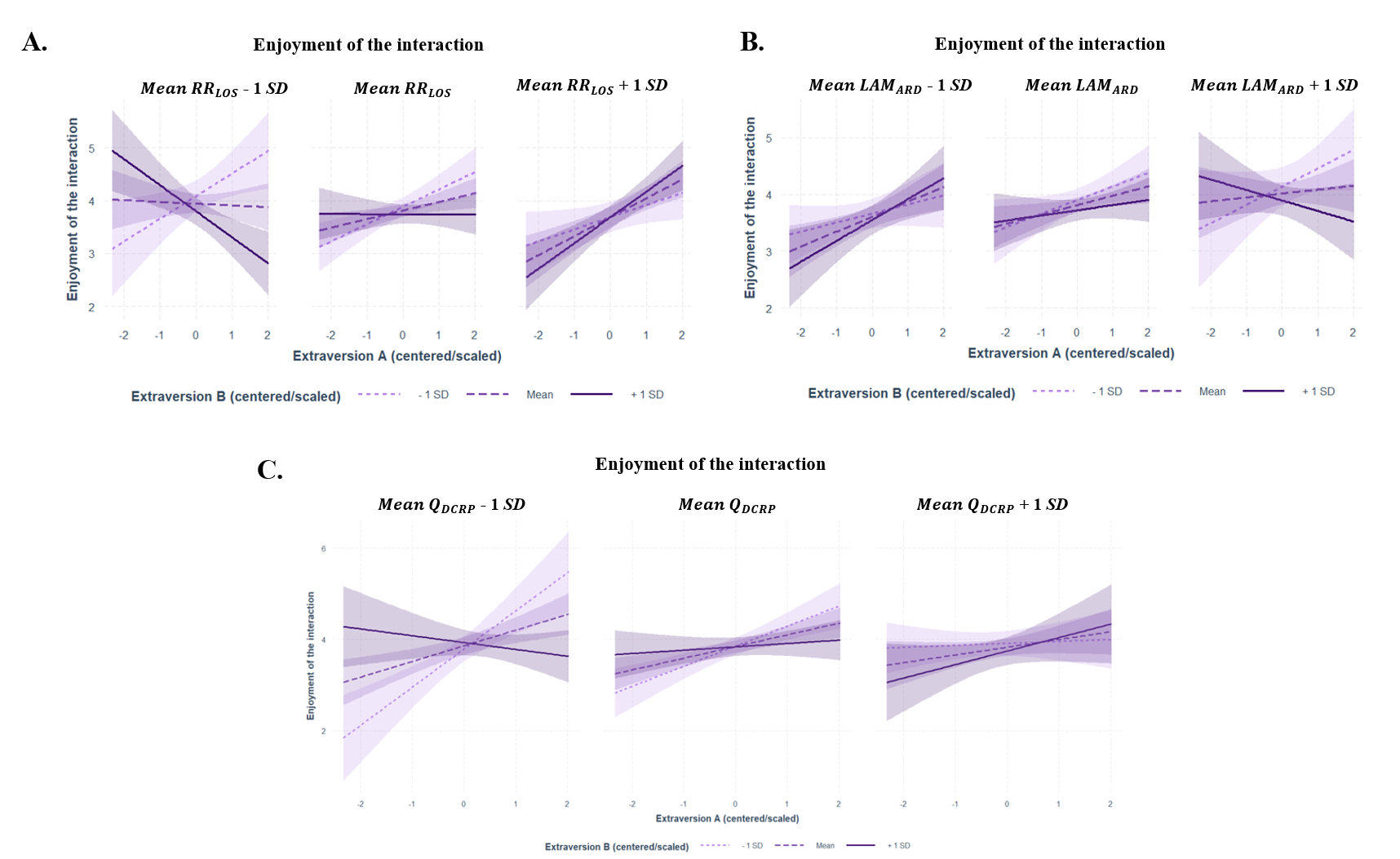


*Note:* Panel A shows the effects of the Extraversion scores of both conversation partners and *RR_LOS_* (-1SD, mean, and +1SD) on the enjoyment of the interaction (*y-axis*) (Table S1, Model 11). Panel B shows the effects of the Extraversion scores of both conversation partners and *LAM_ARD_* (-1SD, mean, and +1SD) on the enjoyment of the interaction (*y-axis*) (Table S2, Model 11). Panel C shows the effects of the Extraversion scores of both conversation partners and *Q_DCRP_* (-1SD, mean, and +1SD) on the enjoyment of the interaction (*y-axis*) (Table S3, Model 11).

**Figure S.6.**

*Liked the other person predicted by Extraversion and RR_LOS_* *_
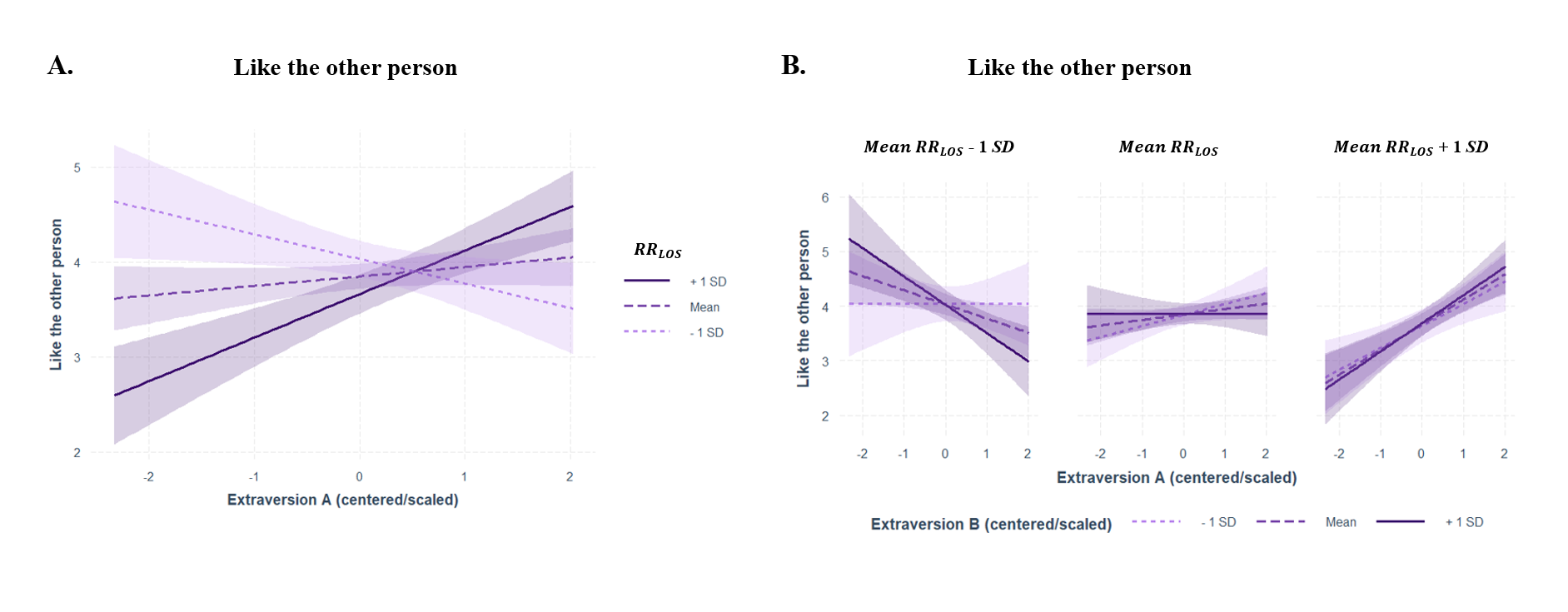
_*

*Note:* Panel A shows the effects of the Extraversion scores of one conversation partner and *RR_LO_*_S_ (-1SD, mean, and +1SD) on the reported liking of the other person (*y-axis*) (Table S1, Model 14). Panel B shows the effects of the Extraversion scores of both conversation partners and *RR_LOS_* (-1SD, mean, and +1SD) on the reported liking of the other person (*y-axis*) (Table S1, Model 14).

**Figure S.7.**

*Perceived partner as empathic and understanding predicted by Extraversion and LAM_ARD_*


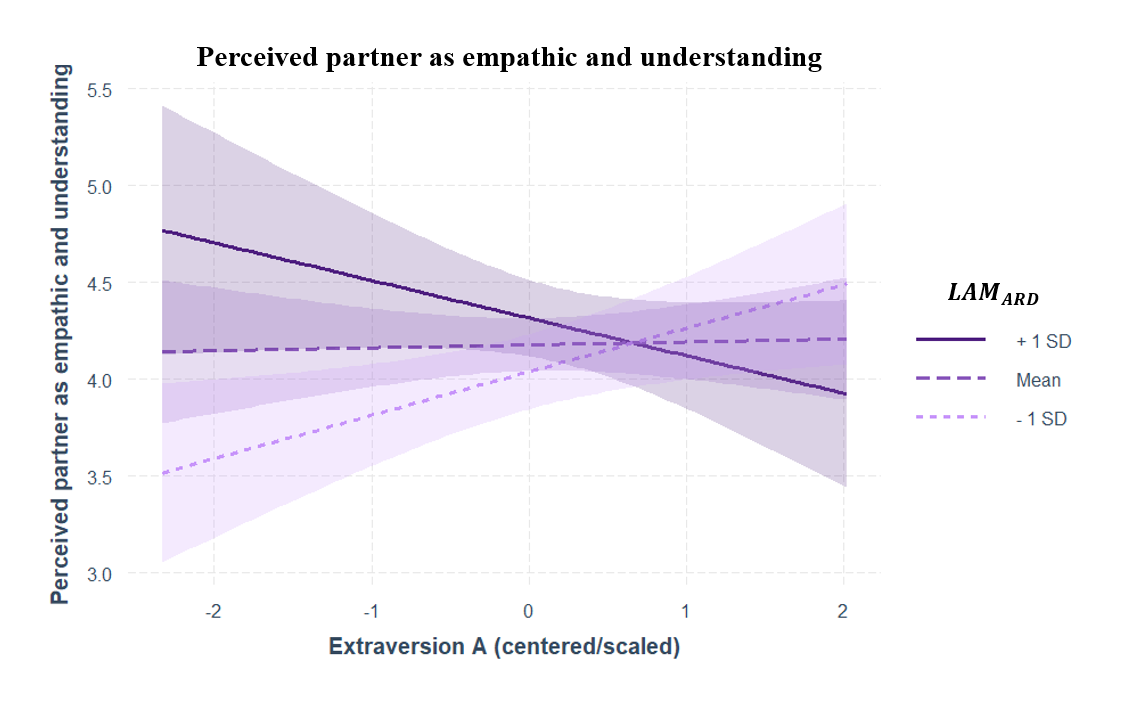


*Note:* The figure shows the effects of the Extraversion scores of one conversation partner and *LAM_ard_* (-1SD, mean, and +1SD) on the reported perception of the partner as empathic and understanding (*y-axis*) (Table S2, Model 15).
