# Supplementary material for: Beyond Words: Speech Coordination Linked to Personality and Appraisals
Source: J Nonverbal Behav. 2025 Mar 8;49(1):85–123. doi: 10.1007/s10919-025-00482-3 (PMC11982161; doi:10.1007/s10919-025-00482-3)
Supplement: Supplementary file 3 — Supplementary file3 (DOCX 47 KB) [file 10919_2025_482_MOESM3_ESM.docx]

**Supplement: Tables**

**Table S.1.**

*General Linear Models predicting Perception of the* Interaction *variables from RR_LoS_* and *Extraversion. N*_i_*=100 participants*

|  | M1. | M2. | M3. | M4. | M5. | M6. | M7. | M8. | M9. | M10. | M11. | M12. | M13. | M14. | M15. |
| --- | --- | --- | --- | --- | --- | --- | --- | --- | --- | --- | --- | --- | --- | --- | --- |
| Predictor | 𝛽 | 𝛽 | 𝛽 | 𝛽 | 𝛽 | 𝛽 | 𝛽 | 𝛽 | 𝛽 | 𝛽 | 𝛽 | 𝛽 | 𝛽 | 𝛽 | 𝛽 |
| Intercept | 0.03*** | .03*** | -0.01*** | -0.01*** | -0.03*** | 0.03*** | 0.00*** | -0.01*** | -0.01*** | -0.02*** | 0.00*** | -0.01*** | -0.00*** | -0.01*** | -0.01*** |
| (E_A_) | -1.24 | -0.07 | 0.17 | 0.04 | -0.18 | 0.25 | 0.10 | -0.08 | 0.26 | -0.02*** | 0.23 | 0.00 | 0.17 | 0.14*** | 0.06 |
| (E_B_) | 2.36* | -0.05 | -0.18 | 0.03 | -0.03 | 0.03 | -0.04 | -0.05 | 0.08 | -0.09 | -0.10 | -0.19 | 0.04 | 0.01 | -0.02 |
| RR_LoS_ | -0.73 | -0.03 | 0.12 | -0.12 | -0.01 | -0.11 | -0.31* | -0.21 | -0.15 | -0.24* | -0.18 | -0.09 | -0.07 | -0.25* | -0.18 |
| E_A_ * E_B_ | -3.22** | -0.15 | 0.14 | -0.04 | 0.19 | -0.26 | -0.10 | 0.11 | 0.02 | -0.14 | -0.23*** | -0.04 | -0.09 | -0.14* | -0.10 |
| E_A_ **RR_LoS_* | 1.74. | -0.28 | -0.16 | -0.01 | -0.08 | 0.10 | 0.26 | 0.05 | 0.17 | 0.46*** | 0.28* | 0.09 | 0.26 | 0.49*** | 0.30 |
| E_B_ * *RR_LoS_* | -2.29* | 0.14 | -0.04 | 0.23 | 0.14 | 0.03 | -0.09 | -0.10 | -0.22 | 0.12 | 0.10 | 0.23 | -0.07 | 0.02 | -0.00 |
| E_A_ * E_B_ * *RR_LoS_* | 3.19** | 0.07 | -0.04 | 0.00 | -0.21 | 0.19 | 0.18 | -0.10 | 0.25 | 0.08 | 0.42*** | -0.04 | 0.25 | 0.21* | 0.17 |
| AIC | 218.07 | 257.66 | 244.50 | 336.89 | 286.50 | 262.91 | 228.14 | 271.69 | 212.42 | 246.28 | 194.28 | 285.85 | 230.59 | 206.48 | 210.09 |

*Note:* 𝛽 = standardized beta weights. ** indicates p < .05. ** indicates p < .01. ***indicates p <.000. p*-values were corrected in the full model by Benjamini-Hochberg procedure (Benjamini & Hochberg, 1995). E_A_=Extraversion participant A, E_B_=Extraversion participant B. ICC = Intra-class Correlation Coefficient. AIC = Akaike’s Information Criterion (lower values indicate better fit). Personality traits were centered and scaled. M1= Inclination for communication; M2= Using partner’s behavior as a guide for own behavior; M3= Attempts to lead the conversation; M4= Feeling self-conscious during the conversation; M5= Conversation seemed awkward, forced and strained; M6= Conversation seemed smooth, natural, and relaxed; M7= Conversation felt involving; M8= Felt put down, patronized or rejected by partner; M9= Felt accepted and respected by partner; M10= Desire to interact more with partner in the future; M11= Enjoyment of the interaction; M12= Tried to accommodate to fit in partner’s behavior; M13= Felt comfortable around the partner; M14= Liked conversation partner; M15= Perceived partner as empathic and understanding.

**Table S.2.**

*General Linear Models predicting Perception of the* Interaction *variables from LAM_ARD_* and *Extraversion. N*_i_*=100 participants*

|  | M1. | M2. | M3. | M4. | M5. | M6. | M7. | M8. | M9. | M10. | M11. | M12. | M13. | M14. | M15. |
| --- | --- | --- | --- | --- | --- | --- | --- | --- | --- | --- | --- | --- | --- | --- | --- |
| Predictor | 𝛽 | 𝛽 | 𝛽 | 𝛽 | 𝛽 | 𝛽 | 𝛽 | 𝛽 | 𝛽 | 𝛽 | 𝛽 | 𝛽 | 𝛽 | 𝛽 | 𝛽 |
| Intercept | 0.01*** | 0.03*** | -0.04*** | 0.02*** | 0.02*** | -0.00*** | -0.00*** | -0.01*** | -0.03*** | 0.04*** | 0.00*** | 0.04*** | -0.01*** | 0.02*** | 0.03*** |
| (E_A_) | 0.20* | -0.14** | 0.18 | 0.02 | -0.22 | 0.28 | 0.09 | -0.07 | 0.25** | 0.00 | 0.23* | -0.04 | 0.20 | 0.16** | 0.02* |
| (E_B_) | 0.04 | -0.08 | -0.14 | 0.03 | -0.04 | 0.04 | -0.09 | -0.07 | 0.03 | -0.12 | -0.13 | -0.21 | 0.01 | -0.03 | -0.09 |
| *LAMa_RDiff_* | 0.12 | -0.36 | -0.07 | 0.06 | -0.16 | 0.23 | 0.25 | -0.14 | 0.12 | 0.13 | 0.29* | 0.04 | 0.14 | 0.20 | 0.21 |
| E_A_ * E_B_ | -0.04 | 0.27* | 0.11 | -0.02 | 0.15 | -0.22 | -0.02 | 0.03 | 0.09 | -0.08 | -0.11 | 0.04 | -0.01 | -0.05 | 0.01 |
| E_A_ * *LAM_ARD_* | -0.20 | -0.18* | 0.11 | 0.10 | -0.04 | 0.08 | -0.17 | -0.05 | -0.22 | -0.18 | -0.14 | -0.06 | -0.14 | -0.27 | -0.31* |
| E_B_ * *LAM_ARD_* | 0.16 | -0.18 | 0.10 | -0.19 | -0.20 | 0.11 | 0.09 | -0.03 | 0.11 | 0.04 | -0.04 | -0.13 | 0.08 | 0.06 | -0.05 |
| E_A_ * E_B_ * *LAM_ARD_* | -0.03 | 0.23* | 0.03 | 0.02 | 0.10 | -0.11 | -0.08 | -0.07 | -0.17 | 0.06 | -0.25* | 0.21 | -0.12 | -0.10 | -0.07 |
| AIC | 231.03 | 249.74 | 245.47 | 336.82 | 283.29 | 257.87 | 234.89 | 76.44 | 219.11 | 260.93 | 206.71 | 285.03 | 237.79 | 224.06 | 211.54 |

*Note:* 𝛽 = standardized beta weights. ** indicates p < .05. ** indicates p < .01. ***indicates p <.000. p*-values were corrected in the full model by Benjamini-Hochberg procedure (Benjamini & Hochberg, 1995). E_A_=Extraversion participant A, E_B_=Extraversion participant B. ICC = Intra-class Correlation Coefficient. AIC = Akaike’s Information Criterion (lower values indicate better fit). Personality traits were centered and scaled. M1= Inclination for communication; M2= Using partner’s behavior as a guide for own behavior; M3= Attempts to lead the conversation; M4= Feeling self-conscious during the conversation; M5= Conversation seemed awkward, forced and strained; M6= Conversation seemed smooth, natural, and relaxed; M7= Conversation felt involving; M8= Felt put down, patronized or rejected by partner; M9= Felt accepted and respected by partner; M10= Desire to interact more with partner in the future; M11= Enjoyment of the interaction; M12= Tried to accommodate to fit in partner’s behavior; M13= Felt comfortable around the partner; M14= Liked conversation partner; M15= Perceived partner as empathic and understanding.

**Table S.3.**

*General Linear Models predicting Perception of the* Interaction *variables from Q_DCRP_* and *Extraversion. N*_i_*=100 participants*

|  | M1. | M2. | M3. | M4. | M5. | M6. | M7. | M8. | M9. | M10. | M11. | M12. | M13. | M14. | M15. |
| --- | --- | --- | --- | --- | --- | --- | --- | --- | --- | --- | --- | --- | --- | --- | --- |
| Predictor | 𝛽 | 𝛽 | 𝛽 | 𝛽 | 𝛽 | 𝛽 | 𝛽 | 𝛽 | 𝛽 | 𝛽 | 𝛽 | 𝛽 | 𝛽 | 𝛽 | 𝛽 |
| Intercept | 0.02*** | -0.01*** | -0.03*** | -0.05*** | -0.04*** | 0.05*** | 0.04*** | -0.02*** | 0.01*** | 0.02** | 0.05*** | -0.00*** | 0.01*** | -0.00*** | -0.00*** |
| (E_A_) | 0.26 | -0.19 | 0.13 | -0.08 | -0.26 | 0.34 | 0.21 | -0.10 | 0.35 | 0.07 | 0.36* | -0.02 | 0.24 | 0.22 | 0.10 |
| (E_B_) | 0.06 | -0.08 | -0.20 | -0.03 | -0.06 | 0.10 | -0.01 | -0.10 | 0.08 | -0.08 | -0.01 | -0.18 | 0.03 | -0.01 | -0.05 |
| *Q_DCRP_* | 0.01 | 0.01 | -0.11 | -0.01 | -0.14 | 0.17 | 0.00 | -0.11 | 0.05 | 0.06 | -0.02 | -0.01 | 0.05 | 0.07 | -0.05 |
| E_A_ * E_B_ | -0.03 | -0.14 | 0.11 | -0.06 | 0.11 | -0.26 | -0.12 | 0.01 | 0.10 | -0.20 | -0.26** | -0.02 | -0.02 | -0.19 | -0.16 |
| E_A_ * *Q_DCRP_* | 0.02 | -0.09 | -0.17 | -0.07 | 0.15 | 0.01 | 0.02 | -0.02 | 0.21 | 0.01 | -0.12 | -0.02 | 0.13 | -0.09 | -0.16 |
| E_B_ * *Q_DCRP_* | 0.13 | -0.14 | 0.06 | -0.27 | 0.02 | -0.07 | -0.03 | -0.15 | -0.07 | -0.20 | -0.11 | 0.05 | -0.14 | -0.27 | -0.16 |
| E_A_ * E_B_ * *Q_DCRP_* | 0.08 | -0.15 | -0.02 | -0.34 | -0.21 | 0.27 | 0.30 | -0.01 | 0.23 | 0.05 | 0.44* | -0.16 | 0.07 | 0.04 | 0.06 |
| AIC | 236.98 | 263.84 | 243.63 | 331.48 | 286.40 | 259.03 | 241.36 | 76.51 | 222.75 | 260.79 | 209.67 | 288.42 | 240.04 | 226.09 | 216.56 |

*Note:* 𝛽 = standardized beta weights. ** indicates p < .05. ** indicates p < .01. ***indicates p <.000. p*-values were corrected in the full model by Benjamini-Hochberg procedure (Benjamini & Hochberg, 1995). E_A_=Extraversion participant A, E_B_=Extraversion participant B. ICC = Intra-class Correlation Coefficient. AIC = Akaike’s Information Criterion (lower values indicate better fit). Personality traits were centered and scaled. M1= Inclination for communication; M2= Using partner’s behavior as a guide for own behavior; M3= Attempts to lead the conversation; M4= Feeling self-conscious during the conversation; M5= Conversation seemed awkward, forced and strained; M6= Conversation seemed smooth, natural, and relaxed; M7= Conversation felt involving; M8= Felt put down, patronized or rejected by partner; M9= Felt accepted and respected by partner; M10= Desire to interact more with partner in the future; M11= Enjoyment of the interaction; M12= Tried to accommodate to fit in partner’s behavior; M13= Felt comfortable around the partner; M14= Liked conversation partner; M15= Perceived partner as empathic and understanding.

**Table S.4.**

*General Linear Models predicting Perception of the* Interaction *variables from RR_LoS_* and *Agreeableness. N*_i_*=100 participants*

|  | M1. | M2. | M3. | M4. | M5. | M6. | M7. | M8. | M9. | M10. | M11. | M12. | M13. | M14. | M15. |
| --- | --- | --- | --- | --- | --- | --- | --- | --- | --- | --- | --- | --- | --- | --- | --- |
| Predictor | 𝛽 | 𝛽 | 𝛽 | 𝛽 | 𝛽 | 𝛽 | 𝛽 | 𝛽 | 𝛽 | 𝛽 | 𝛽 | 𝛽 | 𝛽 | 𝛽 | 𝛽 |
| Intercept | 0.02*** | 0.00*** | -0.05*** | -0.01*** | 0.00*** | -0.00*** | 0.02*** | -0.01*** | 0.01*** | 0.06*** | 0.03*** | 0.04*** | 0.03*** | 0.04*** | 0.01*** |
| (A_A_) | 0.01 | 0.10 | 0.07 | -0.18 | -0.15 | -0.00* | 0.01 | -0.21 | 0.08 | -0.07 | 0.04 | -0.09 | 0.13 | 0.05 | -0.02 |
| (A_B_) | 0.01 | 0.06 | -0.28 | -0.03 | -0.11 | -0.03 | -0.16 | -0.08 | -0.15 | -0.02 | -0.19 | 0.04 | -0.08 | -0.24 | -0.29 |
| *RR_LoS_* | -0.03 | -0.10 | 0.05 | -0.09 | 0.06 | -0.16 | -0.33* | -0.16 | -0.12 | -0.13 | -0.10 | -0.07 | -0.05 | -0.13 | -0.17 |
| A_A_ * A_B_ | -0.15 | 0.01 | 0.12 | -0.03 | 0.09 | -0.12 | -0.15 | 0.15 | -0.05 | -0.14 | -0.18 | -0.13 | -0.15 | -0.16 | -0.16 |
| A_A_ * *RR_LoS_* | -0.05 | 0.04 | -0.08 | -0.08 | 0.28 | -0.35* | -0.09 | 0.19 | -0.05 | -0.09 | -0.16 | 0.20 | -0.13 | -0.05 | -0.13 |
| A_B_ * *RR_LoS_* | 0.07 | -0.10 | -0.04 | -0.09 | 0.06 | -0.07 | -0.10 | 0.16 | 0.13 | 0.17 | 0.06 | -0.24 | -0.03 | 0.02 | -0.16 |
| A_A_ * E_B_ * *RR_LoS_* | -0.21 | 0.10 | -0.09 | -0.01 | -0.16 | 0.15 | 0.09 | -0.20 | -0.10 | 0.15 | -0.01 | 0.17 | 0.14 | 0.01 | -0.02 |
| AIC | 235.92 | 267 | 241.39 | 337.99 | 284.94 | 269.32 | 236.30 | 59.12 | 232.79 | 261.15 | 224.21 | 285.08 | 242.86 | 232.40 | 211.15 |

*Note:* 𝛽 = standardized beta weights. ** indicates p < .05. ** indicates p < .01. ***indicates p <.000. p*-values were corrected in the full model by Benjamini-Hochberg procedure (Benjamini & Hochberg, 1995). E_A_=Extraversion participant A, E_B_=Extraversion participant B. ICC = Intra-class Correlation Coefficient. AIC = Akaike’s Information Criterion (lower values indicate better fit). Personality traits were centered and scaled. M1= Inclination for communication; M2= Using partner’s behavior as a guide for own behavior; M3= Attempts to lead the conversation; M4= Feeling self-conscious during the conversation; M5= Conversation seemed awkward, forced and strained; M6= Conversation seemed smooth, natural, and relaxed; M7= Conversation felt involving; M8= Felt put down, patronized or rejected by partner; M9= Felt accepted and respected by partner; M10= Desire to interact more with partner in the future; M11= Enjoyment of the interaction; M12= Tried to accommodate to fit in partner’s behavior; M13= Felt comfortable around the partner; M14= Liked conversation partner; M15= Perceived partner as empathic and understanding.

**Table S.5.**

*General Linear Models predicting Perception of the* Interaction *variables from LAM_ARD_* and *Agreeableness. N*_i_*=100 participants*

|  | M1. | M2. | M3. | M4. | M5. | M6. | M7. | M8. | M9. | M10. | M11. | M12. | M13. | M14. | M15. |
| --- | --- | --- | --- | --- | --- | --- | --- | --- | --- | --- | --- | --- | --- | --- | --- |
| Predictor | 𝛽 | 𝛽 | 𝛽 | 𝛽 | 𝛽 | 𝛽 | 𝛽 | 𝛽 | 𝛽 | 𝛽 | 𝛽 | 𝛽 | 𝛽 | 𝛽 | 𝛽 |
| Intercept | 0.04*** | 0.03*** | -0.03*** | 0.01*** | 0.00*** | -0.01*** | 0.03*** | -0.02*** | 0.01*** | 0.04*** | 0.03*** | 0.06*** | 0.00*** | 0.03*** | 0.03*** |
| (A_A_) | -0.05 | -0.02 | 0.05 | -0.24 | -0.13 | -0.02 | -0.02 | -0.18 | 0.09 | -0.11 | 0.01 | -0.15 | 0.17 | 0.01 | -0.05 |
| (A_B_) | -0.01 | -0.11 | -0.33* | -0.09 | -0.09 | -0.02 | -0.19 | -0.06 | -0.11 | -0.04 | -0.20 | -0.10 | -0.04 | -0.26 | -0.32 |
| *LAM_ARD_* | 0.05 | 0.14 | -0.05 | 0.08 | -0.16 | 0.28* | 0.29* | -0.13 | 0.13 | 0.16 | 0.31* | 0.04 | 0.13 | 0.23 | 0.19 |
| A_A_ * A_B_ | -0.08* | 0.35 | 0.19 | 0.13 | 0.03 | -0.08 | 0.00 | 0.11 | -0.11 | -0.10 | -0.18 | 0.13 | -0.25 | -0.12 | -0.02 |
| A_A_ * *LAM_ARD_* | -0.09 | -0.02 | 0.18 | -0.03 | -0.19 | 0.16 | -0.06 | 0.06 | 0.00 | 0.05 | 0.12 | -0.13 | 0.12 | -0.02 | -0.06 |
| A_B_ * *LAM_ARD_* | 0.11 | -0.33* | -0.15 | -0.04 | 0.12 | 0.00 | -0.04 | -0.05 | 0.07 | -0.14 | -0.06 | -0.21 | 0.05 | 0.02 | 0.02 |
| A_A_ * A_B_ * *LAM_ARD_* | 0.16 | 0.27* | 0.14 | 0.16 | -0.02 | 0.00 | 0.04 | 0.04 | -0.05 | -0.03 | -0.02 | 0.16 | -0.13 | 0.01 | 0.10 |
| AIC | 233.45 | 257.16 | 236.10 | 336.32 | 284.15 | 266.41 | 238.16 | 71.53 | 234.65 | 262.27 | 214.62 | 286.97 | 240.93 | 228.46 | 210.98 |

*Note:* 𝛽 = standardized beta weights. ** indicates p < .05. ** indicates p < .01. ***indicates p <.000. p*-values were corrected in the full model by Benjamini-Hochberg procedure (Benjamini & Hochberg, 1995). E_A_=Extraversion participant A, E_B_=Extraversion participant B. ICC = Intra-class Correlation Coefficient. AIC = Akaike’s Information Criterion (lower values indicate better fit). Personality traits were centered and scaled. M1= Inclination for communication; M2= Using partner’s behavior as a guide for own behavior; M3= Attempts to lead the conversation; M4= Feeling self-conscious during the conversation; M5= Conversation seemed awkward, forced and strained; M6= Conversation seemed smooth, natural, and relaxed; M7= Conversation felt involving; M8= Felt put down, patronized or rejected by partner; M9= Felt accepted and respected by partner; M10= Desire to interact more with partner in the future; M11= Enjoyment of the interaction; M12= Tried to accommodate to fit in partner’s behavior; M13= Felt comfortable around the partner; M14= Liked conversation partner; M15= Perceived partner as empathic and understanding.

**Table S.6.**

*General Linear Models predicting Perception of the* Interaction *variables from Q_DCRP_* and *Agreeableness. N*_i_*=100 participants*

|  | M1. | M2. | M3. | M4. | M5. | M6. | M7. | M8. | M9. | M10. | M11. | M12. | M13. | M14. | M15. |
| --- | --- | --- | --- | --- | --- | --- | --- | --- | --- | --- | --- | --- | --- | --- | --- |
| Predictor | 𝛽 | 𝛽 | 𝛽 | 𝛽 | 𝛽 | 𝛽 | 𝛽 | 𝛽 | 𝛽 | 𝛽 | 𝛽 | 𝛽 | 𝛽 | 𝛽 | 𝛽 |
| Intercept | 0.02*** | -0.02*** | -0.07*** | -0.01*** | 0.01*** | -0.01 | 0.01*** | -0.03*** | 0.01*** | 0.05*** | 0.04*** | 0.01*** | 0.04*** | 0.04*** | 0.02*** |
| (A_A_) | -0.04 | 0.08 | -0.07 | -0.19 | -0.14 | -0.02 | 0.02 | -0.21 | 0.08 | -0.03 | 0.04 | -0.12 | 0.18 | 0.07 | 0.01 |
| (A_B_) | -0.01 | 0.03 | -0.37 | -0.04 | -0.11 | -0.02 | -0.15 | -0.10 | -0.15 | 0.02 | -0.16 | -0.03 | -0.03 | -0.20 | -0.27 |
| *Q_DCRP_* | 0.01 | 0.07 | -0.14 | -0.07 | -0.17 | 0.20 | 0.06 | -0.08 | 0.05 | 0.07 | 0.05 | -0.01 | 0.03 | 0.07 | -0.02 |
| A_A_ * A_B_ | -0.09 | 0.13 | 0.17* | -0.06 | -0.00 | 0.01 | 0.02 | 0.25 | 0.05 | -0.22 | -0.12 | -0.03 | -0.20 | -0.17 | -0.03 |
| A_A_ * *Q_DCRP_* | -0.02 | 0.07 | -0.03 | -0.15 | 0.15 | -0.12 | 0.02 | 0.07 | -0.01 | -0.11 | 0.02 | 0.12 | -0.04 | 0.04 | 0.03 |
| A_B_ * *Q_DCRP_* | -0.10 | -0.05 | -0.34* | -0.11 | -0.07 | -0.02 | 0.04 | 0.16 | 0.14 | 0.10 | 0.03 | -0.14 | 0.02 | -0.05 | 0.05 |
| A_A_ * A_B_ * *Q_DCRP_* | -0.20 | -0.13 | -0.32* | -0.07 | 0.08 | -0.15 | -0.11 | -0.11 | -0.10 | 0.15 | -0.03 | 0.13 | 0.15 | 0.04 | -0.06 |
| AIC | 236.52 | 266.75 | 226.98 | 337.28 | 285.71 | 272.96 | 244.81 | 69.39 | 234.697 | 261.84 | 226.28 | 287.57 | 243 | 233.07 | 216.13 |

*Note:* 𝛽 = standardized beta weights. ** indicates p < .05. ** indicates p < .01. ***indicates p <.000. p*-values were corrected in the full model by Benjamini-Hochberg procedure (Benjamini & Hochberg, 1995). E_A_=Extraversion participant A, E_B_=Extraversion participant B. ICC = Intra-class Correlation Coefficient. AIC = Akaike’s Information Criterion (lower values indicate better fit). Personality traits were centered and scaled. M1= Inclination for communication; M2= Using partner’s behavior as a guide for own behavior; M3= Attempts to lead the conversation; M4= Feeling self-conscious during the conversation; M5= Conversation seemed awkward, forced and strained; M6= Conversation seemed smooth, natural, and relaxed; M7= Conversation felt involving; M8= Felt put down, patronized or rejected by partner; M9= Felt accepted and respected by partner; M10= Desire to interact more with partner in the future; M11= Enjoyment of the interaction; M12= Tried to accommodate to fit in partner’s behavior; M13= Felt comfortable around the partner; M14= Liked conversation partner; M15= Perceived partner as empathic and understanding.
